# Pre‐Regenerative Endothelial Cells Empowered by Antler Blood Exosomes Orchestrate Peripheral Nerve Repair

**DOI:** 10.1002/advs.202522170

**Published:** 2026-08-27

**Authors:** Jinsheng Huang, Qiang Chen, Aihua Jin, Xiaoqi Yang, Hui Zhou, Weiquan Gong, Jin Cheng, Jun Dai, Minjin Wang, Meihua Piao, Yuanyi Wang, Nan Zhou

**Affiliations:** ^1^ Department of Orthopedics The First Affiliated Hospital of Zhengzhou University Zhengzhou University Zhengzhou China; ^2^ Department of Sports Medicine of the Second Affiliated Hospital and Liangzhu Laboratory Zhejiang University School of Medicine Hangzhou China; ^3^ Department of Clinical Laboratory Yanbian University Hospital Yanji China; ^4^ Department of Spinal Surgery Orthopedics Center, The First Hospital of Jilin University Jilin University Changchun China; ^5^ Department of Sports Medicine Beijing Key Laboratory of Sports Injuries Peking University Third Hospital Beijing China; ^6^ Department of Orthopedic Surgery The Second Affiliated Hospital of Soochow University Suzhou China; ^7^ Department of Laboratory Medicine West China Hospital of Sichuan University Chengdu China

**Keywords:** antler blood, exosomes, IMP3, niche, peripheral nerve regeneration, pre‐regenerative endothelial cells

## Abstract

The pre‐regenerative vascular niche (PVN) is indispensable for peripheral‐nerve repair, yet the endothelial cell (EC) blueprint underlying PVN formation remains to be fully defined. Herein, we mapped the sciatic‐nerve endothelium and identified angiogenic ECs (AECs) as the dominant pre‐regenerative subset triggered by injury. However, the molecular circuitry governing AEC‐mediated regeneration awaits elucidation. Unexpectedly, we found that antler blood, routinely discarded during velvet antler harvesting, contains potent regenerative cues that strongly boost endothelial‐driven repair. By integrating a single‐cell atlas of antler with the proteomics of antler blood–derived exosomes (AB‐EXO), we traced the vesicles to their cellular origins and demonstrated that AB‐EXO encapsulate the antler's potent regenerative signature. Further mechanistic interrogation showed that AB‐EXO induced pro‐angiogenic phenotypes in ECs via the upregulation of IMP U3 small nucleolar ribonucleoprotein 3 (IMP3). Overlaying IMP3 expression onto our EC atlas pinpointed IMP3‐high AECs (IMP3+AECs) as the pivotal vascular drivers of nerve repair. Ultimately, our findings establish IMP3+AECs as the critical component of the pre‐regenerative EC atlas within the peripheral nerve and position AB‐EXO as an effective therapy for nerve repair.

## Graphical abstract

The pre‐regenerative vascular niche (PVN) is essential for nerve repair, yet its endothelial blueprint remains unclear. We identified angiogenic ECs (AECs) as the dominant pre‐regenerative subset and found that antler blood–derived exosomes (AB‐EXO) promote repair via IMP3. AB‐EXO induces pro‐angiogenic IMP3+AECs as critical PVN drivers, establishing AB‐EXO as effective therapy.

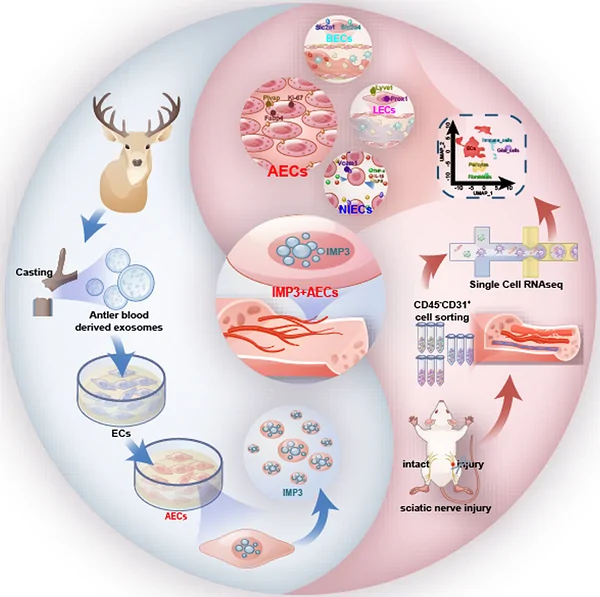

## 1 Introduction

In peripheral nerve injury (PNI), disruption of the local vasculature and breakdown of the blood‐nerve barrier constitute a universal pathological hallmark, whereas effective tissue repair depends on robust endothelial cell (EC) regeneration to reconstruct a functionally integrated vascular network [1]. Early angiogenesis furnishes the regenerative vascular niche with both the metabolic fuel required for swift nerve repair and the angiogenic cues that vectorially guide Schwann cell (SC) migration [2]. In our recent study, we initially analyzed the anatomical structure and pro‐regenerative mechanisms of the pre‐regenerative vascular niche (PVN) in peripheral nerve, and subsequently established NTN1‐engineered EC exosomes (NTN1 EC‐EXO) to evoke the formation of PVN [3]. As regenerative medicine matures, tissue‐specific ECs are no longer seen as passive vascular linings but as gatekeeping master regulators of organogenesis, homeostasis, and disease [4, 5]. By acquiring unique phenotypes in distinct vascular beds, they deploy a stereotyped angiocrine repertoire that drives morphogenesis, sustains homeostasis, and directs regeneration [6, 7]. The molecular circuitry that orchestrates angiogenesis in the peripheral nerve remains only partially mapped, yet it is poised to reveal the organotypic identity of ECs and their crosstalk with niche‐resident partners [8]. Single‐cell cartography revealed how ECs self‐organize into a polarized micro‐scaffold at the lesion site of injured peripheral nerves. This precisely choreographed repair angiogenesis erects a “bridge” microenvironment that simultaneously shepherds regenerating axons and safeguards the long‐term homeostasis of the newly formed tissue [9].

Although the molecular circuitry guiding EC–mediated regeneration in peripheral nerve has been dissected, an effective therapeutic paradigm remains conspicuously absent—spurring the urgent quest to harness the EC subpopulation endowed with the strong regenerative potency. We therefore turned our attention to a singular exemplar of regenerative medicine: the deer antler—the only mammalian organ capable of complete, cyclic regeneration [10]. Antler regrowth is driven not only by the resident mesenchymal and periosteal stem cells that harbor prodigious self‐renewal and chondro‐osteogenic potency, but also by the systemic milieu delivered through antler blood [11]. Recently, the single‐cell atlas of antler regrowth unveiled a unique mesenchymal stem cell (MSC) population—antler blastema progenitor cells (ABPCs), with impressive capacities for self‐renewal and tissue repair [12]. Subsequent work revealed that ABPC‐derived extracellular vesicles (ABPC‐EVs) act as potent geroprotective agents, blunting cellular senescence phenotypes and revitalizing both skeletal and systemic aging organs [13]. Yet ABPC‐EVs are presently isolated from a single stem‐cell line, divorced from the broader pro‐regenerative milieu of the antler in which osteoblasts, endothelial cells, pericytes, fibroblasts, and immune cells jointly sculpt the regenerative niche. EVs harvested directly from tissues or blood are presumed to reflect a more comprehensive and substantive signature; we therefore turned to antler blood—a source that is abundant, ethically sustainable, and physiologically authentic [14, 15]. Furthermore, antler blood was abundant in systemic bioactive factors, providing a nurturing and conductive environment for antler growth and regeneration, while the supply of this blood was abundant and readily sustainable, ensuring broad applicative potential [16, 17]. However, the exact cellular origin of AB‐EXO remains undefined, leaving their relationships with the diverse cell types of antler tissue obscure. Then we integrated antler scRNA‐seq data with the AB‐EXO proteomics to pinpoint the cellular origin of AB‐EXO and demonstrated that AB‐EXO encapsulate the antler's potent regenerative program.

ECs from injured peripheral nerves have limited regenerative ability, curtailing the angiogenic drive required for robust neural repair. Therefore, identifying innovative ECs with strong regenerative potential might refine the role of ECs in peripheral‐nerve regeneration. Lessons from repair angiogenesis during injured nerve regeneration may suggest strategies to ensure neovascularization and exploit the instructive action of ECs on driving nerve repair in clinical settings. The molecular blueprint governing angiogenesis within the peripheral nervous system remains fragmentary, largely because ECs constitute only a minute fraction of the total nerve cellularity. To resolve how the vascular niche is re‐established after injury, we performed scRNA‐seq on ECs purified from rat sciatic nerves and assembled the comprehensive endothelial atlas of the peripheral nerve. This approach uncovered a discrete, injury‐responsive EC subpopulation—angiogenic ECs (AECs)—that undergoes rapid clonal expansion following PNI. Leveraging the robust pro‐regenerative capacity of AB‐EXO, we performed quantitative proteomic profiling of treated ECs and identified IMP U3 small nucleolar ribonucleoprotein 3 (IMP3) as their critical molecular effector. AB‐EXO elicit a marked up‐regulation of IMP3, unleashing a pro‐angiogenic program that instructs the assembly of a regeneration‐competent vascular niche. This function was corroborated by complementary gain‐of‐function studies: lentiviral over‐expression in vitro and AAV‐mediated delivery in vivo both notably enhanced endothelial proliferation and sprouting. Integrating IMP3 expression into our single‐cell atlas further resolved IMP3‐high AECs (IMP3+AECs) as the principal vascular drivers orchestrating peripheral‐nerve repair. Our data establish IMP3+AECs as the dominant architects of post‐injury neovascularization and nominate AB‐EXO as a potent, IMP3‐directed therapeutic for harnessing this specialized endothelial subpopulation to accelerate nerve regeneration.

## 2 Results

### 2.1 ScRNA‐Seq Atlas of EC Heterogeneity in the Rat Sciatic Nerve

**FIGURE 1:**
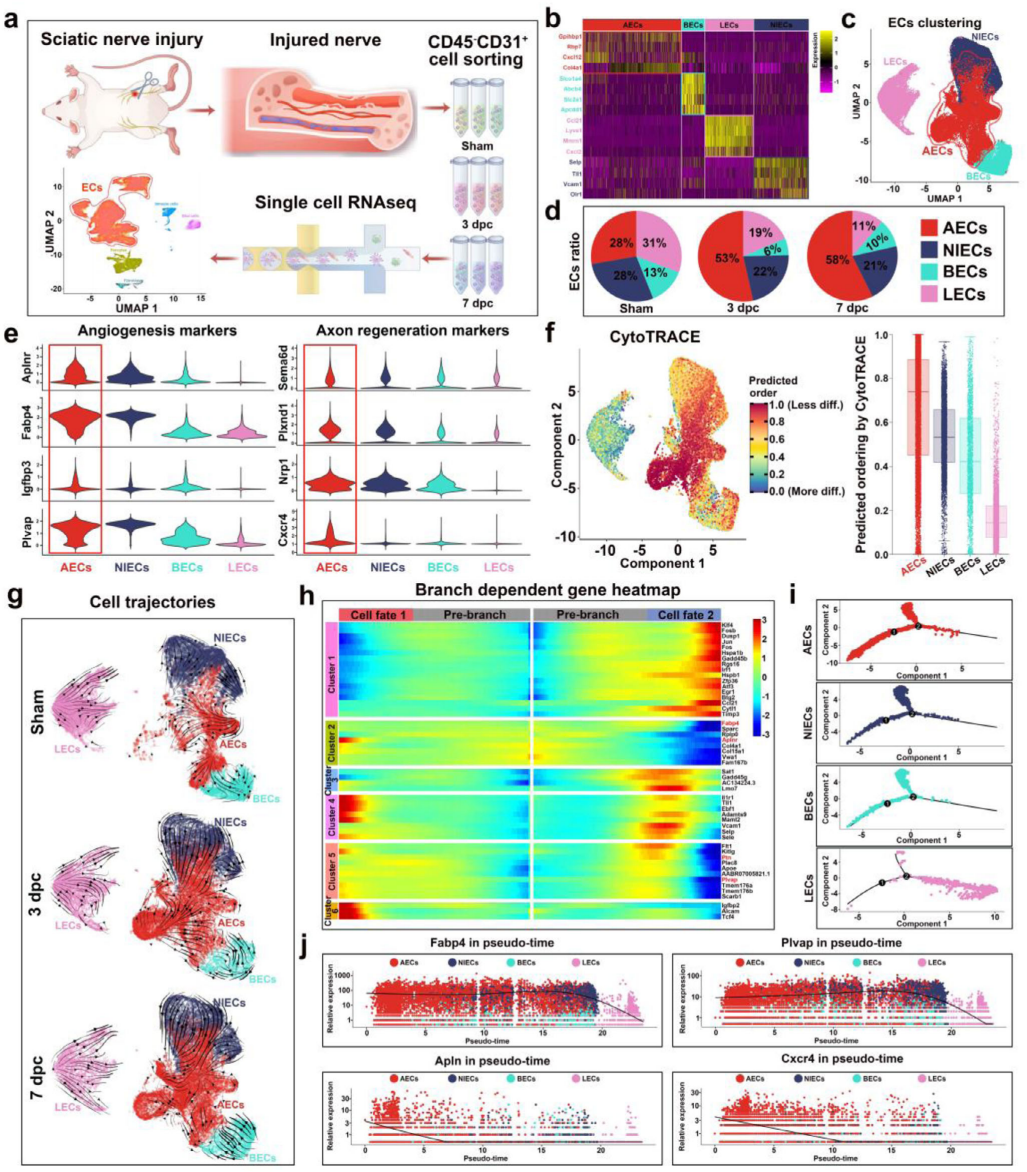
ScRNA‐seq revealed an atlas of ECs in sciatic nerve. (a) Schematic of ECs FACS‐isolation from the sciatic nerve and scRNA‐seq. (b) Heatmap showing EC class‐level marker gene expression. (c) Uniform manifold approximation and projection (UMAP) atlas of color‐coded EC classes involved in nerve regeneration based on the scRNA‐seq data. (d) Relative abundance of EC subtypes in sham, 3‐ and 7‐dpc groups. (e) Violin plots showing distribution of angiogenesis markers and axon regeneration markers in different EC clusters. (f) CytoTRACE analysis for AECs, NIECs, BECs, and LECs. (g) RNA velocity vectors superimposed on UMAP plot with different EC clusters. (h) Heatmap showing the expression dynamic of genes with different patterns along the two branches, with the genes categorized into six clusters via hierarchical clustering. (i) EC trajectories in integrated scRNA‐seq dataset color‐coded by cell‐type composition. (j) Pseudo‐time kinetics of representative genes.

To further dissect the formation of the vascular niche during peripheral nerve regeneration, we performed unilateral sciatic‐nerve crush in adult rats and harvested nerves at 3 and 7 days post crush (dpc); contralateral, uninjured nerves served as sham controls [3]. Then CD31^+^CD45^−^ ECs were subsequently FACS‐purified from intact and injured nerves and subjected to scRNA‐seq. In addition to ECs, CD31^+^CD45^−^ cell populations in sciatic nerve also included pericytes, glial cells, immune cells and fibroblasts (Figure 1a and Figure S1). Unsupervised clustering of these ECs resolved nineteen transcriptionally distinct subtypes (Figure S1). Leveraging the previous studies and the expression profiles of top‐ranking subtype‐specific markers, we further consolidated the nineteen EC subtypes into four functionally coherent EC clusters [9, 18, 19]. Among these ECs, the angiogenic ECs (AECs), comprising clusters 1, 2, 3, 7, 9, 12, and 16, selectively expressed pro‐regenerative markers; the neuroinflammatory ECs (NIECs), represented by clusters 4, 6, 8, and 11, were distinguished by selectively expressed inflammatory markers; the barrier ECs (BECs), represented by clusters 5 and 13, were distinguished by selectively expressed blood‐tissue barrier markers; the lymphatic ECs (LECs), comprising clusters 0, 10, 14, 15, 17, and 18, selectively expressed lymphatic EC markers (Figure 1b,c and Figure S1). Post injury, AECs increased notably, whereas the remaining three clusters‐ NIECs, BECs and LECs were progressively diminished; barrier‐forming BECs registered the sharpest decline (Figure 1d and Figure S1). Moreover, AECs robustly expressed angiogenesis markers (Aplnr, Fabp4, Igfbp3 and Plvap) and axon regeneration markers (Sema6d, Plxnd1, Nrp1, and Cxcr4), underscoring their pivotal regenerative capacity during nerve repair (Figure 1e).

Cell cycle profiling (G2/M and S phase scores) revealed pronounced proliferative activity selectively within AECs, indicating that these cells are actively engaged in DNA replication and preparing for mitosis (Figure S2). GO and KEGG enrichment analysis further delineated the distinct molecular landscapes and signaling pathways that define AECs, NIECs, BECs, and LECs (Figure S2). CytoTRACE analysis revealed that AECs exhibit the lowest differentiation state among all EC clusters, positioning them at a less‐mature, more plastic endpoint of the continuum (Figure 1f). To dissect the injury‐responsive role of AECs, we generated heatmaps and violin plots that chart the differentially expressed genes (DEGs) across sham, 3 dpc and 7 dpc nerves, highlighting key regulators of angiogenesis, exosome biogenesis, nerve repair, and axon regeneration. The results indicated that AECs executed a pronounced transcriptional switch, markedly up‐regulating a suite of pro‐regenerative molecules that orchestrate vascular remodeling and axonal regrowth (Figure S3). Complementary GO‐term and KEGG‐enrichment analyses further delineated the stage‐specific molecular landscapes and signaling networks activated within AECs across sham, 3 dpc and 7 dpc nerves (Figure S3). Next, trajectory analysis of differentiation revealed limited transitions among EC clusters in the sham group, whereas a pronounced exchange was evident in both 3 and 7 dpc groups. More intriguingly, NIECs were predicted to differentiate into AECs within injured nerves, thereby accounting for the post‐injury expansion of the AECs (Figure 1g–i). We further tracked the pseudo‐temporal dynamics of representative genes—Fabp4, Plvap, Apln, and Cxcr4—to capture their regulatory trajectories across the EC differentiation continuum (Figure 1j). Then we employed CellChat analysis to comprehensively map the EC‐EC communication atlas. CellChat quantified the probability of communication via specific ligand–receptor (L‐R) pairs (Figure S4). CellChat elucidated how distinct EC clusters and their cognate signaling molecules orchestrate intercellular communication through incoming and outgoing communication patterns (Figure S4). CellChat further resolved numerous L‐R pairs among EC subpopulations and classified them into distinct signaling networks (Figure S4). We next leveraged CellChat to dissect angiogenic signaling, identifying Angpt and Angptl signaling pathways as the prominent pathways. Within these pathways, we systematically quantified the contribution of each EC cluster as sender, receiver, mediator, or influencer (Figure S4). Further interrogation of the Angpt/Angptl circuitry uncovered a marked enrichment of pro‐angiogenic L‐R pairs post injury (Figure S4). Collectively, our data demonstrated that the peripheral‐nerve vasculature exhibited marked molecular–functional heterogeneity, and that injury reprogramed this heterogeneous endothelium, culminating in a prominent pro‐regenerative state.

### 2.2 AB‐EXO Encapsulated the Strong Regenerative Potential of Antler

**FIGURE 2:**
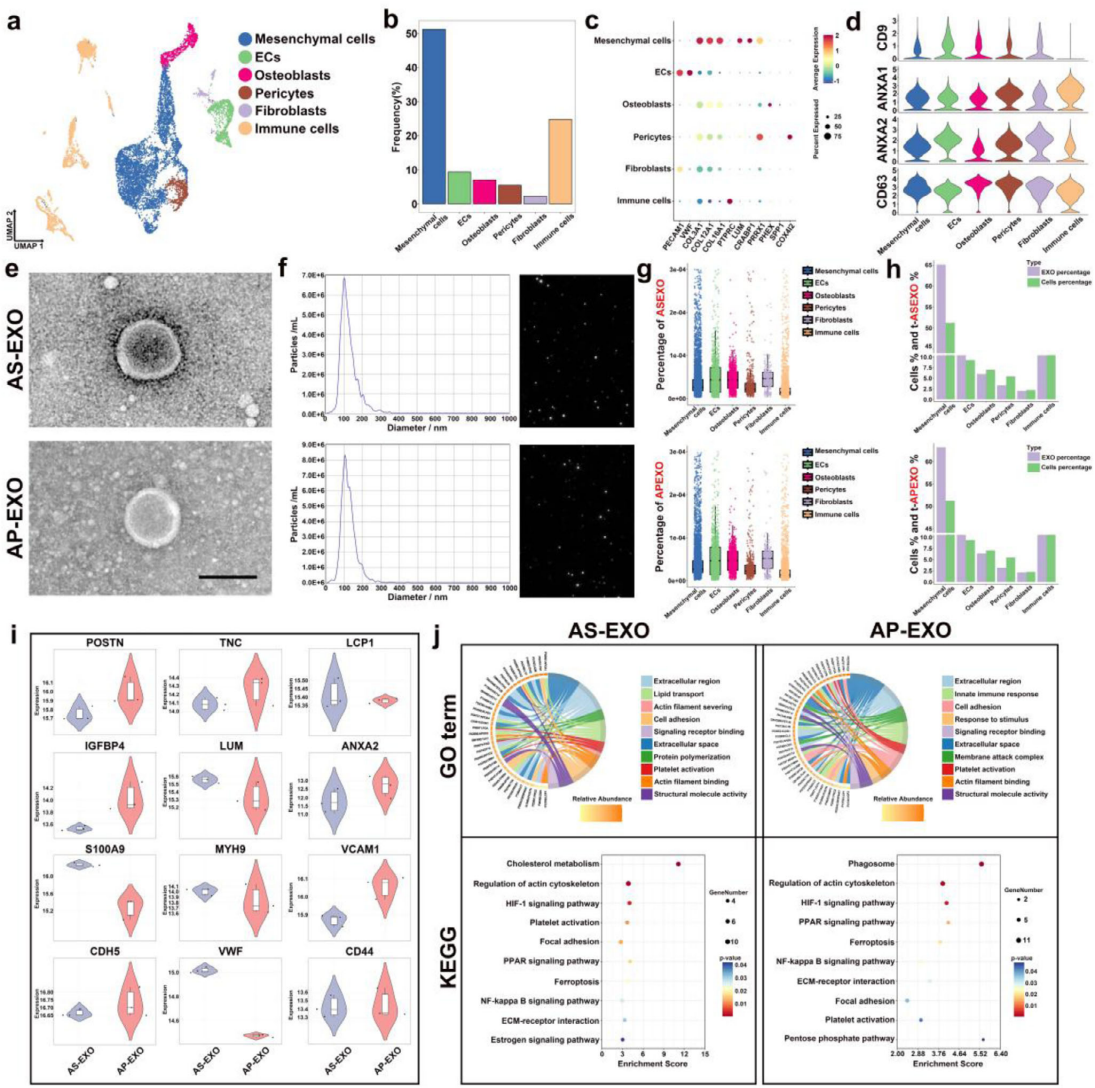
Analysis of antler scRNA‐seq data together with EXO proteomics identified that AB‐EXO could carry the strong regenerative capacity of antler via EXO tracing. (a) UMAP atlas of color‐coded cell classes involved in antler regeneration. (b) Proportions of different cell types. (c) Dot plot showing cell class‐level marker gene expression. (d) Violin plots displaying the expression levels of representative genes associated with EXO‐secretion in different cell clusters. (e) Representative TEM images of AS‐EXO and AP‐EXO. Scale bar, 100 nm. (f) Size distribution of AS‐EXO and AP‐EXO detected by NTA, inset showing representative images captured from the NTA video frames. (g) Percentage of AS‐EXO and AP‐EXO from each single cell, shown as the classification of cell cluster. (h) Proportion of each cluster among total cells, and proportion of AS‐EXO and AP‐EXO contributed by each cluster. (i) Violin plots showing the expression of regeneration‐related proteins in AS‐EXO and AP‐EXO. (j) GO term and KEGG enrichment analysis of specifically expressed proteins in AS‐EXO and AP‐EXO.

Current EC‐rejuvenation strategies are still searching for a definitive blueprint. Exploiting the deer antler—the only cyclic organ in nature, we obtained blood‐derived EXO (AB‐EXO) that capture the full regenerative orchestra, compared to the soloist profile of ABPC‐EVs. By cross‐mapping antler scRNA‐seq with proteomes of antler serum (AS‐EXO) and plasma (AP‐EXO) vesicles, we traced the proteins back to their cellular source and gauged each cell's vesicle output [20]. We thank Qin et al. for publicly releasing their scRNA‐seq dataset, which we leveraged to survey the robust regenerative phase of antler observed five days after casting [12]. Cells from antler were visualized so that they could be assigned to different cell populations, including mesenchymal cells, ECs, osteoblasts, pericytes, fibroblasts, and immune cells (Figure 2a). We then quantified the relative abundance of each population and profiled its defining marker genes. Among these, mesenchymal cells emerged as the predominant population with high‐expression levels of LUM and PRRX1 (Figure 2b,c). Then we examined markers of EXO biogenesis and export across antler cell types, underscoring the functional importance of EXO release during antler regeneration (Figure 2d). AS‐EXO and AB‐EXO were isolated and rigorously characterized by morphology, size distribution, and proteomics. Transmission electron microscopy (TEM) revealed both populations as round vesicles enclosed by a classic lipid bilayer membrane (Figure 2e). Nanoparticle tracking analysis (NTA) revealed nearly identical size distributions, with AS‐EXO averaging 101.2 nm and AP‐EXO 103.7 nm in diameter (Figure 2f). Moreover, we have now performed Western blotting for both positive markers (CD63, CD81, TSG101) and negative marker (Calnexin) to further strengthen the characterization of AB‐EXO (Figure S5). Protein profiling was then used to evaluate the individual ability of single cells to release EXO. Quantitative profiling revealed mesenchymal cells as the predominant EXO secretors, identifying them as the principal source of both AS‐EXO and AP‐EXO. Furthermore, ECs formed the second‐largest EXO pool, underscoring EXO secretion as a key mechanism by which ECs drive antler regeneration (Figure 2g). Integrating scRNA‐seq and proteomic data revealed that mesenchymal cells and ECs collectively produce the vast majority of AS‐EXO and AP‐EXO (Figure 2h). Proteomic profiling also showed that both AS‐EXO and AP‐EXO were enriched in antler‐associated bioactive factors (Figure 2i). GO and KEGG enrichment analysis further resolved the molecular signatures and signaling pathways of AS‐EXO and AP‐EXO (Figure 2j). Thus, both AS‐EXO and AP‐EXO encapsulated the strong regenerative potential of antlers, positioning AB‐EXO as a remarkable therapeutic strategy for regenerative medicine.

### 2.3 AB‐EXO Facilitated Angiogenesis In Vitro

**FIGURE 3:**
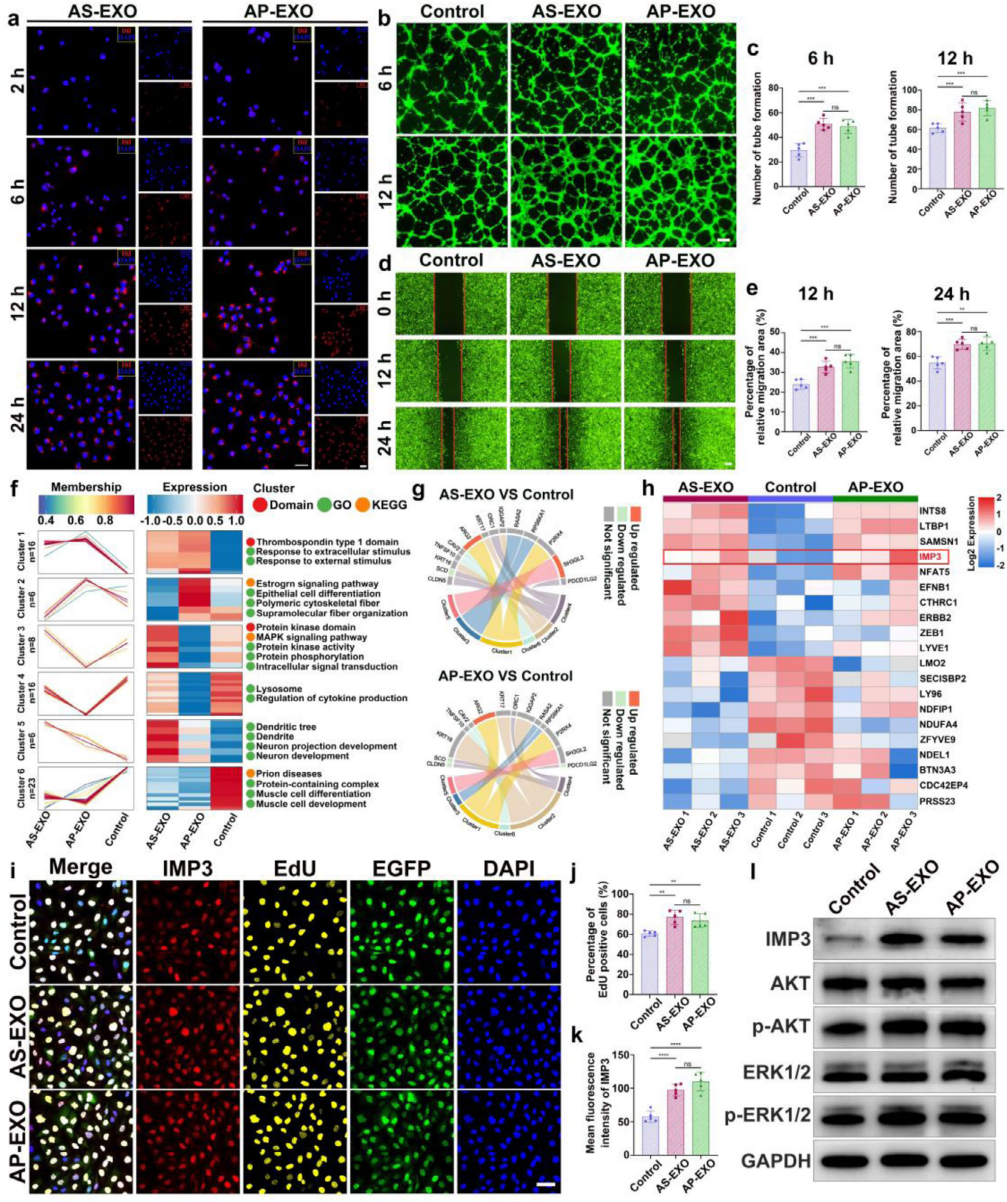
AB‐EXO boosted angiogenesis‐related phenotypes of ECs and up‐regulated IMP3 expression. (a) Representative fluorescence images of ECs at 2, 6, 12 and 24 h following co‐culture with DiI‐labeled EXO. Scale bar, 50 µm. (b,c) Tube formation ability of ECs with each treatment at 6 and 12 h. Scale bar, 200 µm. (d,e) Wound healing rate of ECs with each treatment after 12 and 24 h. Scale bar, 200 µm. (f) Differentially expressed proteins (DEPs) from all groups were combined into a single union set and categorized into six distinct clusters. Domain, GO, and KEGG enrichment analysis were performed for proteins within each cluster. (g) Chord diagrams depicting the distribution of DEPs across clusters among different groups. (h) Heatmap showing DEPs among different groups. (i) Representative EdU and IMP3 staining images in different groups. Scale bar, 50 µm. (j) Statistical evaluation of percentage of EdU‐positive ECs. (k) Statistical analysis of mean fluorescence intensity of IMP3. (l) Western blot detecting the expression of IMP3, AKT, p‐AKT, ERK1/2, p‐ERK1/2 in different groups. ns = not significant, **p < 0.01, ***p < 0.001, ****p < 0.0001.

Rapid antler regeneration is accompanied by the rapid expansion of vasculature. Then we evaluated the effect of AS‐EXO and AP‐EXO on angiogenesis in vitro. Internalized DiI‐labeled EXO accumulated in the perinuclear region of ECs, confirming their active uptake and potential functional impact (Figure 3a). As compared to the control group (treated with PBS), both AS‐EXO and AP‐EXO markedly enhanced tubular maturation and junctional density, with no appreciable difference between the two EXO groups (Figure 3b,c). Additionally, both AS‐EXO and AP‐EXO notably elevated the proportion of ECs in the S phase when compared with the control group (Figure S5). Treatment with either AS‐EXO or AP‐EXO markedly accelerated EC migration, yielding notably more transmembrane cells than the PBS control (Figure S5). Scratch‐wound assays further demonstrated that, relative to the control, both AS‐EXO and AP‐EXO markedly accelerated EC migration, as evidenced by the substantially larger wound‐closure area (Figure 3d,e). We next corroborated the pro‐proliferative activity of AS‐EXO and AP‐EXO using the colony‐formation assay. ECs exposed to either AS‐EXO or AP‐EXO generated a notably greater number of colonies, underscoring their capacity to enhance clonogenic expansion (Figure S6). Furthermore, we also isolated exosomes from bovine serum (BS‐EXO) as additional controls. Functional comparisons in migration and tube formation assays demonstrate that AB‐EXO exhibit significantly superior pro‐angiogenic activity compared to BS‐EXO, confirming that the observed regenerative effects are specific to the rapid‐growth antler state rather than foreign nanoparticle effects (Figure S7).

### 2.4 Proteomic Analysis of ECs Treated with AS‐EXO and AP‐EXO

Having established that both AS‐EXO and AP‐EXO potentiate angiogenesis in vitro, we next performed quantitative proteomics on control, AS‐EXO–treated, and AP‐EXO–treated ECs to map the key protein networks underlying this pro‐angiogenic phenotype. The differentially expressed proteins (DEPs) from each group were collected and converted into a union set. The fuzzy C‐means (FCM) algorithm is a cornerstone unsupervised clustering technique in the field of data mining [21]. We subsequently applied the fuzzy C‐means algorithm to partition the fold‐change data from all three groups into six functionally distinct proteomic clusters. Cluster 1 and cluster 5 showed that AS‐EXO and AP‐EXO groups had distinct proteomic characteristics, including response to extracellular stimulus, neuron projection development, and neuron development (Figure 3f and Figure S8). We then generated circus plots to visualize the proteins assigned to each cluster between different groups (Figure 3g). A comparative heatmap of DEPs across control, AS‐EXO, and AP‐EXO groups was constructed, providing an intuitive visualization of the signature proteins that drive the distinct angiogenic phenotypes. Among these proteins, IMP3 emerged as a prominent up‐regulated target in both AS‐EXO‐ and AP‐EXO‐treated ECs (Figure 3h). IMP3 is a well‐recognized guardian of stem‐cell identity and function, exerting its effects through the tight regulation of ribosome biogenesis [22]. We next constructed a protein–protein interaction (PPI) network that mapped the relationships between IMP3 and the other proteins, thereby revealing the molecular partnerships through which IMP3 exerts its pro‐angiogenic effects (Figure S8). To corroborate the proteomic findings, we performed dual‐label immunofluorescence for IMP3 and 5‐ethynyl‐2′‐deoxyuridine (EdU). This allowed simultaneous quantification of IMP3 abundance and the fraction of EdU‐positive ECs following AS‐EXO and AP‐EXO treatment. The results confirmed that both AS‐EXO and AP‐EXO substantially elevated IMP3 expression and markedly augmented EC proliferative capacity (Figure 3i–k). Western blot corroborated that AS‐EXO and AP‐EXO markedly up‐regulated IMP3 expression and concurrently triggered phosphorylation of the pro‐angiogenic kinases AKT and ERK1/2 (Figure 3l). These findings established that AB‐EXO drive angiogenesis through the selective up‐regulation of IMP3, positioning IMP3 as a pivotal molecular target for deciphering—and ultimately harnessing—the regenerative programs of ECs.

### 2.5 IMP3‐High Angiogenic ECs (IMP3+AECs) Constituted the Strong Regenerative Vascular Atlas Within Peripheral Nerve

**FIGURE 4:**
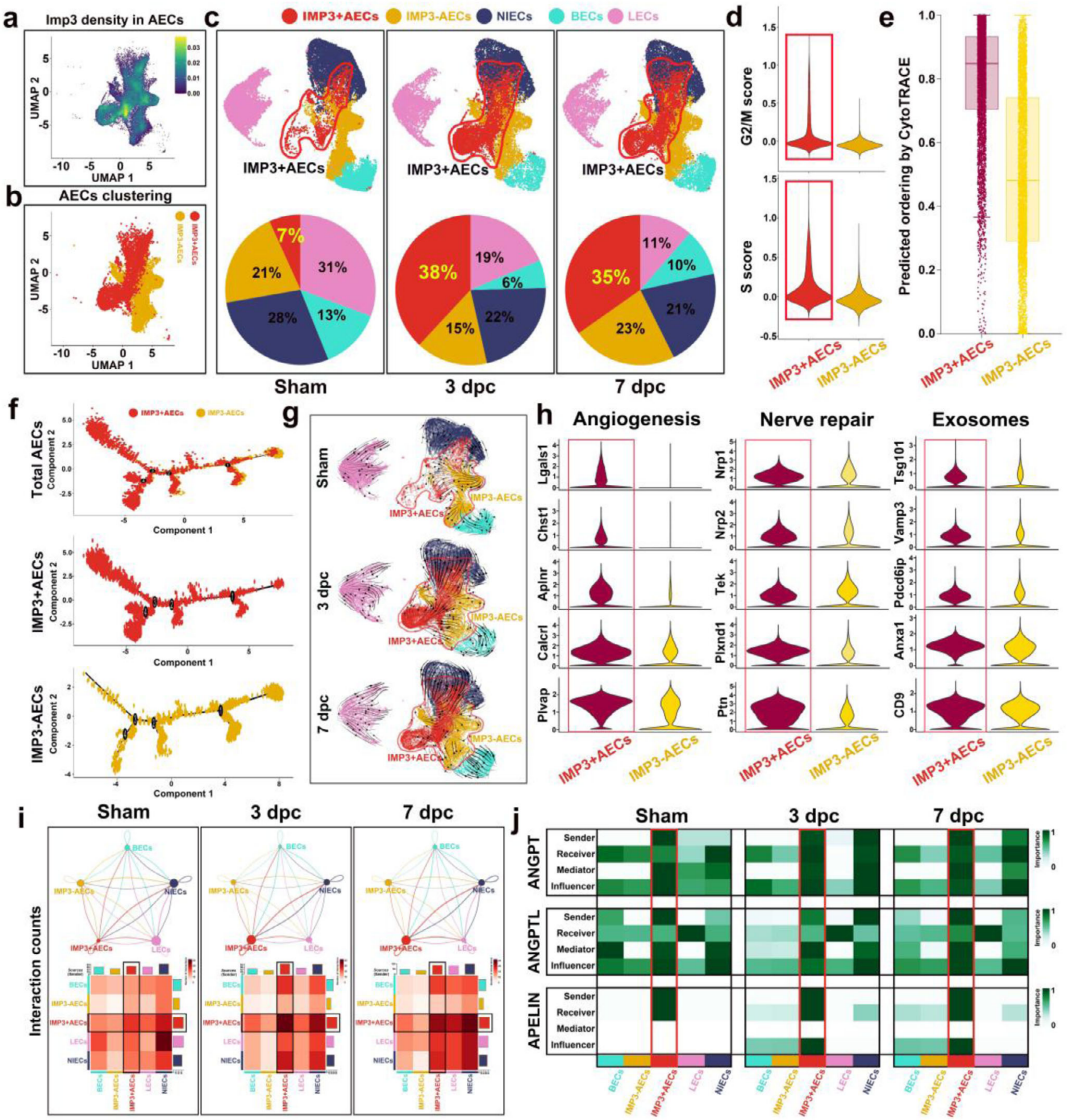
IMP3+AECs constituted a remarkable EC subpopulation with exceptional regenerative capacity within peripheral nerve. (a) Relative density of IMP3 in AECs. (b) UMAP displaying the distribution of IMP3+AECs and IMP3‐AECs. (c) Proportions of each EC cluster in different groups. (d) Seurat clustering analysis of G2/M and S scores in IMP3+AECs and IMP3‐AECs. (e) CytoTRACE analysis for IMP3+AECs and IMP3‐AECs. (f) Cell trajectories for IMP3+AECs and IMP3‐AECs. (g) RNA velocity vectors superimposed on UMAP plot with color‐coded EC clusters. (h) Violin plots showing distribution of angiogenesis, nerve repair, and exosomal markers in IMP3+AECs and IMP3‐AECs. (i) Network and heatmap plots depicting the counts of cell interactions in different groups. (j) Heatmaps illustrating the distinct contributions of each EC cluster to the ANGPT, ANGPTL, and APELIN signaling pathways across different groups.

Having identified IMP3 as the pivotal molecular driver of angiogenesis, we next interrogated its relationship with AECs at single‐cell resolution through scRNA‐seq. Mapping IMP3 density onto the UMAP revealed both the prevalence and spatial distribution of IMP3+AECs within the single‐cell landscape. Then we divided AECs into IMP3+AECs, comprising clusters 1, 7, 9, and 16, and IMP3‐low AECs (IMP3‐AECs), comprising clusters 2, 3, and 12, based on relative IMP3 expression (Figure 4a,b and Figure S9). DEGs between IMP3+AECs and IMP3‐AECs were visualized in a heatmap, revealing the transcriptional signatures that distinguish these two endothelial subpopulations (Figure S9). To interrogate the injury‐responsive dynamics of IMP3+AECs, we quantified their distribution and relative abundance against IMP3‐AECs in sham, 3 dpc and 7 dpc cohorts. Compared with uninjured nerves, injured tissue exhibited a pronounced surge in IMP3+AECs (Figure 4c). GO‐term and KEGG enrichment analyses unveiled the unique molecular landscapes and signaling circuitry that demarcate IMP3+AECs from IMP3‐AECs (Figure S9). Cell‐cycle profiling revealed that IMP3+AECs exhibit markedly elevated proliferative activity compared with IMP3‐AECs, evidenced by robust DNA replication and active preparation for mitosis (Figure 4d). CytoTRACE analysis positioned IMP3+AECs at a markedly less‐differentiated, more plastic state along the developmental continuum, indicating their heightened regenerative potential relative to the IMP3‐AECs (Figure 4e and Figure S10). We then charted the pseudotemporal expression of Ptn, Plxnd1, Cxcr4, and Plvap, revealing their stepwise regulatory trajectories along the AEC differentiation continuum (Figure S10). Cell‐trajectory analysis resolved the lineage relationships and pseudo‐temporal ordering of AECs (Figure 4f and Figure S10). Trajectory inference further indicated that, following injury, NIECs were predicted to differentiate into IMP3+AECs rather than adopt the IMP3‐AECs (Figure 4g). Compared with IMP3‐AECs, IMP3+AECs displayed markedly elevated expression of angiogenic, neuro‐reparative, and exosomal markers, underscoring their central role in orchestrating nerve regeneration (Figure 4h). We next used CellChat to quantify the probability that ECs communicate via specific ligand–receptor pairs (Figure S11). CellChat further dissected the intercellular signaling circuitry, revealing how EC subpopulations—including IMP3+AECs, IMP3‐AECs, NIECs, BECs, and LECs—coordinate bidirectional communication via distinct incoming and outgoing ligand–receptor patterns (Figure S11). CellChat further deconvolved the counts of cell interactions in different groups (Figure 4i). Leveraging CellChat, we next deconstructed the pro‐angiogenic communication landscape and pinpointed the Angpt, Angptl, and Apelin pathways as dominant signaling axes. Within each axis, we systematically quantified the roles—sender, receiver, mediator, or influencer—played by every EC cluster (Figure 4j). Further interrogation of the Angpt/Angptl/Apelin circuitry uncovered a marked enrichment of pro‐angiogenic L‐R pairs post injury (Figure S11). To dissect the transcriptional circuitry governing IMP3+AECs, we employed NicheNet to infer L‐R pairs and downstream ligand‐target regulatory links from our scRNA‐seq data [23]. We first delineated the predicted ligand expression landscape uniquely associated with IMP3+AECs in different groups (Figure S12). We next mapped ligand‐to‐target regulatory potency and quantified the prior interaction likelihood for each NicheNet‐predicted ligand–receptor pair (Figure S12). Collectively, these analyses establish IMP3+AECs as a potent, regeneration‐competent vascular atlas that orchestrates peripheral nerve repair through precisely defined ligand–receptor and ligand–target networks.

### 2.6 IMP3 Induced Angiogenic Phenotypes of ECs In Vitro

**FIGURE 5:**
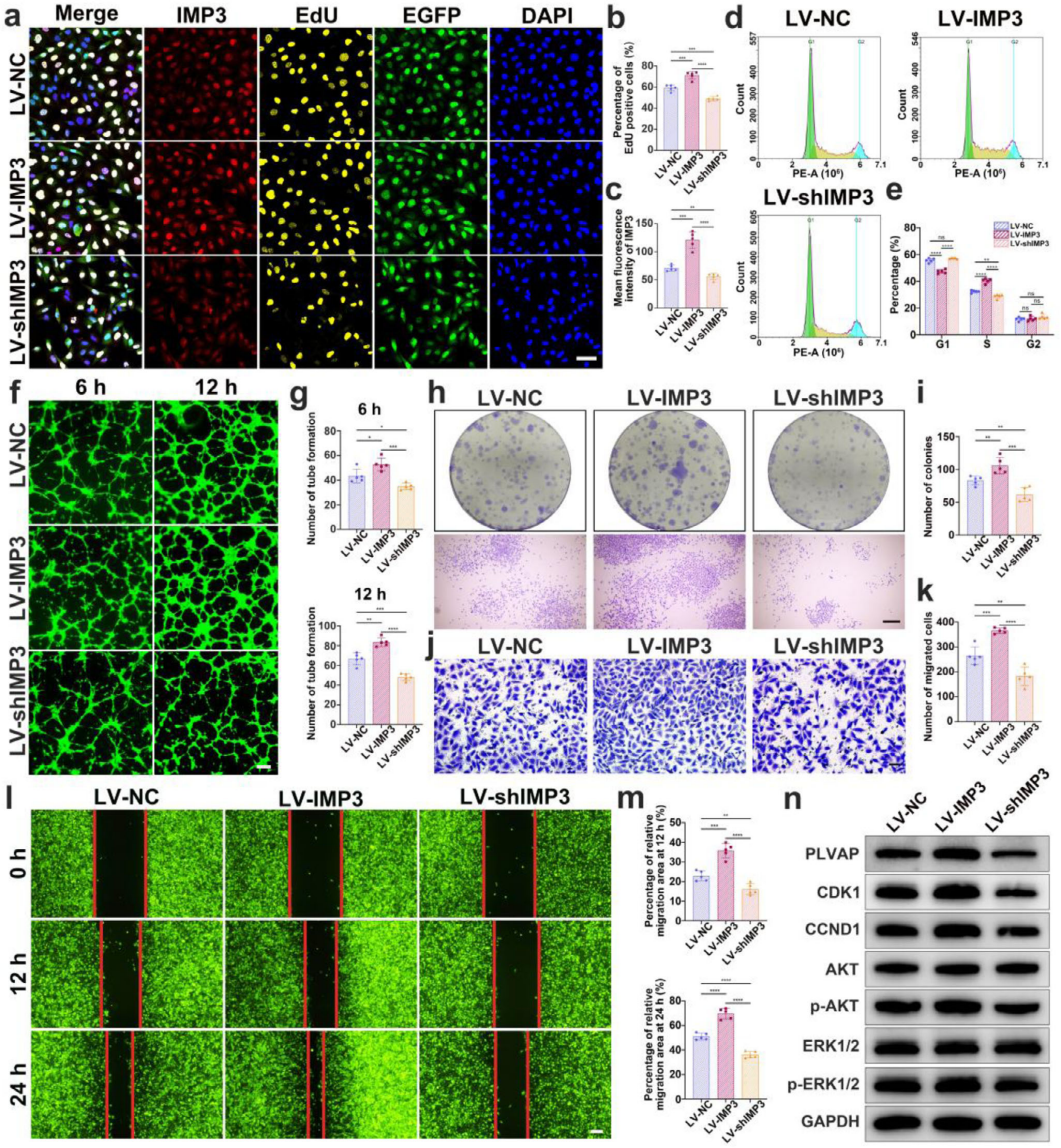
IMP3 promoted angiogenesis in vitro. (a) Representative EdU and IMP3 staining images in different groups. Scale bar, 50 µm. (b,c) Statistical results of the percentage of EdU‐positive ECs and mean fluorescence intensity of IMP3. (d,e) Cell‐cycle profiling by flow cytometry with statistical summary. (f,g) Tube‐formation capacity of ECs with statistical summary. Scale bar, 200 µm. (h,i) Representative images of colony formation with statistical analysis. Scale bar, 500 µm. (j,k) Analysis of EC migration capacity using transwell assay. Scale bar, 50 µm. (l,m) Wound healing rate of ECs with statistical summary. Scale bar, 200 µm. (n) Western blot detecting the expression of PLVAP, CDK1, CCND1, AKT, p‐AKT, ERK1/2, p‐ERK1/2 in different groups. *p < 0.05, **p < 0.01, ***p < 0.001, ****p < 0.0001.

To dissect the role of IMP3 in endothelial angiogenesis, we stably transduced ECs with lentiviral vectors to generate IMP3‐overexpressing (LV‐IMP3), IMP3‐knockdown (LV‐shIMP3), and corresponding negative‐control (LV‐NC) cell lines for in vitro functional assays. Transfection efficiency was rigorously quantified by qPCR for IMP3 mRNA levels and confirmed at the protein level by Western blot (Figure S13). Next, dual‐label immunofluorescence for IMP3 and EdU was performed to map the spatial correlation between IMP3 abundance and EdU‐positive ECs in different groups (Figure 5a). Compared with LV‐NC controls, LV‐IMP3 ECs exhibited markedly elevated IMP3 levels concomitant with a higher EdU‐positive index, whereas LV‐shIMP3 ECs displayed the opposite trend—both IMP3 abundance and EdU incorporation were notably diminished (Figure 5b,c). Flow‐cytometric cell‐cycle profiling showed that IMP3 overexpression expanded the S‐phase population, whereas IMP3 knockdown contracted it, relative to LV‐NC–transduced ECs (Figure 5d,e). Compared with LV‐NC controls, LV‐IMP3 ECs formed markedly more mature, highly interconnected tubules with increased junctional density, whereas LV‐shIMP3 ECs displayed a pronounced reduction in both parameters (Figure 5f,g). The colony‐formation assay further corroborated the pro‐proliferative role of IMP3: LV‐IMP3 ECs yielded notably more and larger colonies than LV‐NC controls, whereas LV‐shIMP3 cells exhibited a marked reduction in both colony number and size (Figure 5h,i). Transwell assays showed that IMP3 overexpression markedly accelerated EC migration, yielding a substantially higher number of transmembrane cells, whereas IMP3 knockdown suppressed this motility (Figure 5j,k). Scratch‐wound assays corroborated these findings: LV‐IMP3 ECs closed the wound markedly faster, achieving a substantially larger closure area than LV‐NC controls, whereas LV‐shIMP3 cells exhibited a pronounced delay in wound closure (Figure 5l,m). Guided by our scRNA‐seq data and prior reports on angiogenic ECs, we quantified representative markers—PLVAP (tumor EC signature) and the cell‐cycle regulators CDK1 and CCND1—by Western blot [24, 25]. We subsequently examined IMP3‐dependent modulation of the pro‐angiogenic AKT and ERK1/2 pathways by assessing their phosphorylation status via Western blot [26]. IMP3 overexpression elevated PLVAP, CDK1, and CCND1 protein levels and simultaneously triggered robust phosphorylation of AKT and ERK1/2, underscoring its role in driving angiogenic signaling (Figure 5n).

To investigate the role of IMP3 in AB‐EXO‐mediated effects, LV‐NC ECs and LV‐shIMP3 ECs were subsequently co‐cultured with AB‐EXO, and functional outcomes including proliferation and migration were systematically examined. The results demonstrated that IMP3 silencing significantly attenuates the pro‐regenerative effects of AB‐EXO, supporting that IMP3 is a critical downstream effector of AB‐EXO‐mediated repair (Figure S14). Moreover, we also performed IMP3 knockdown in ECs and assessed AB‐EXO‐mediated effects in co‐culture with nerve cells. For co‐culture experiments, we utilized a transwell system and conditioned medium preparation based on well‐established protocols from the literature [27]. These results demonstrated that IMP3 silencing significantly abrogates AB‐EXO‐induced pro‐neurogenic effects, confirming that IMP3 is functionally required—rather than merely serving as a marker—for PVN formation and nerve repair (Figure S15). CellChat and NicheNet analyses identified ANGPT/ANGPTL signaling as a prominent mediator of EC‐derived beneficial effects. ANGPTL4, a multifunctional regulator of angiogenesis, inflammation, and vascular permeability, has been targeted therapeutically using neutralizing antibodies in rheumatoid arthritis [28]. TEK, the receptor for ANGPT2, drives angiogenesis through vascular remodeling and can be inhibited by rebastinib, a selective small‐molecule TEK kinase inhibitor that blocks pathways unresponsive to Ang‐sequestering biologics [29]. Guided by these bioinformatics predictions and established pharmacological strategies, we explored anti‐ANGPTL4 antibody and rebastinib in co‐culture systems to investigate IMP3+AEC‐regulated target genes. These results indicated that both anti‐ANGPTL4 antibody and rebastinib attenuate EC‐mediated pro‐regenerative effects (Figure S16 and Movies S1–S6). In summary, ECs over‐expressing IMP3 exhibited a robust angiogenic phenotype in vitro, characterized by accelerated proliferation, directional migration, and capillary‐like tube formation, collectively indicating that IMP3 is a potent driver of neo‐angiogenesis.

### 2.7 IMP3 Accelerated Angiogenesis and Nerve Repair In Vivo

**FIGURE 6:**
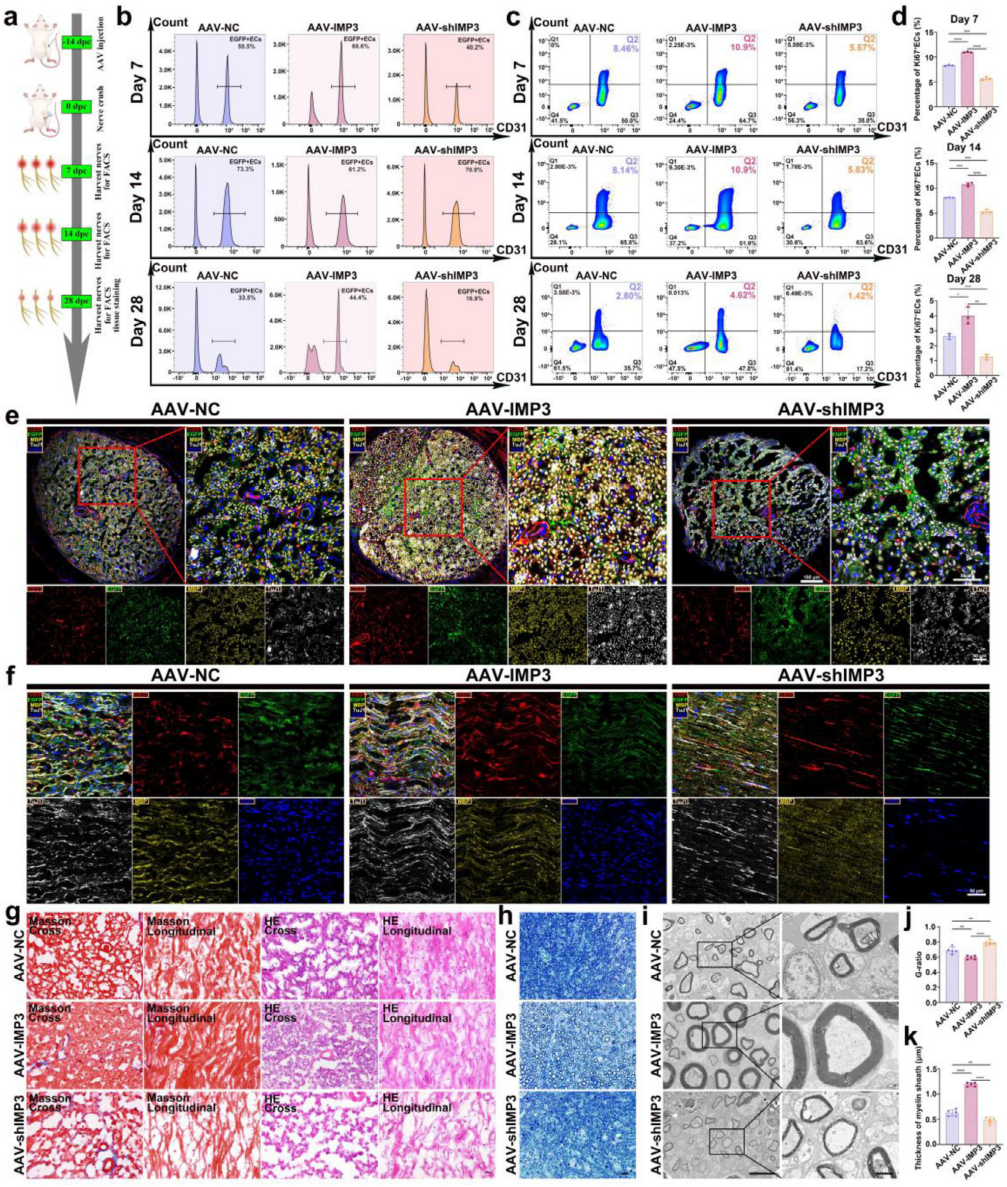
IMP3 accelerated angiogenesis and nerve repair in vivo after injury. (a) The schematic outlines the integrated timeline of AAV delivery, PNI induction, and subsequent tissue‐collection milestones. (b) Percentages of EGFP^+^ ECs quantified by flow cytometry at 7, 14, and 28 days after PNI. (c,d) Percentages of Ki67^+^ ECs quantified by flow cytometry with statistical summary (*n* = 3). (e) Representative five‐color immunofluorescence images (CD31/EGFP/TuJ1/MBP/DAPI) of sciatic‐nerve cross‐sections. Scale bar, 100 and 50 µm. (f) Representative immunofluorescence images of longitudinal sciatic‐nerve sections. Scale bar, 50 µm. (g) Representative H&E‐ and Masson‐stained longitudinal and cross‐sections of sciatic nerves at 28 days post‐surgery. Scale bar, 20 µm. (h) Representative TB‐stained sections of the sciatic nerve for each group. Scale bar, 20 µm. (i) Representative TEM images of sciatic nerve in each group. Scale bar, 10 and 2 µm. (j,k) Statistical analysis of G‐ratio and the thickness of myelin sheath (*n* = 5). *p < 0.05, **p < 0.01, ***p < 0.001, ****p < 0.0001.

To interrogate the angiogenic and neuro‐reparative functions of IMP3 in vivo, we constructed the EC‐specific adenovirus‐associated virus (AAV) in which IMP3 expression is under transcriptional control of the TIE promoter, thereby restricting modulation to ECs within peripheral nerves. Under the sciatic epineurium, rats received a single micro‐injection of either AAV‐NC, AAV‐IMP3 (overexpression), or AAV‐shIMP3 (knockdown). As shown in Figure S17, vascular regeneration precedes axonal regeneration during the early injury phase and subsequently guides nerve repair. Furthermore, AAV‐mediated upregulation of IMP3 in ECs specifically promotes early vascular generation, thereby accelerating axonal regeneration; conversely, IMP3 knockdown in ECs produces the opposite effect (Figure S17). After a 14‐day transduction period, the sciatic nerves were crush‐injured; nerves were then harvested at defined post‐injury intervals for downstream analyses (Figure 6a). The EGFP^+^ cells and EGFP^+^ ECs were quantified by flow cytometry at 7, 14, and 28 days post‐injury (Figure 6b and Figure S18). Compared with the AAV‐NC group, AAV‐IMP3‐transfected injured nerves exhibited a markedly higher fraction of Ki67^+^ ECs, indicative of expanded proliferative vasculature, whereas AAV‐shIMP3 markedly curtailed this response (Figure 6c,d). To capture the interplay among IMP3‐driven angiogenesis, axonal regrowth, and remyelination, we subjected longitudinal and transverse sciatic‐nerve sections to five‐color immunofluorescence (CD31/EGFP/TuJ1/MBP/DAPI) at 28 days post injury (Figure 6e,f). Hematoxylin–eosin (HE) and Masson trichrome staining of cross‐ and longitudinal sciatic‐nerve sections were used to survey nerve architecture and document injury‐induced histopathological alterations. Representative images illustrated that AAV‐IMP3‐treated nerves displayed markedly preserved architecture and minimal vacuolation relative to AAV‐NC controls, whereas AAV‐shIMP3‐treated nerves exhibited the opposite phenotype (Figure 6g). Toluidine‐blue (TB) staining and TEM further revealed that AAV‐IMP3 nerves displayed markedly thicker myelin sheaths and the lower G‐ratio relative to AAV‐NC controls, whereas AAV‐shIMP3 nerves exhibited the opposite profile, thinner myelin and an elevated G‐ratio, underscoring the remarkable role of IMP3 in promoting remyelination (Figure 6h–k and Figure S18). Taken together, our findings established endothelial IMP3 as a potent orchestrator of angiogenesis, axonal regeneration, and remyelination, underscoring targeted IMP3 overexpression in nerve‐resident ECs as a promising therapeutic strategy for peripheral‐nerve repair.

### 2.8 AB‐EXO Exhibited Powerful Pro‐Regenerative Capacity for Nerve Regeneration

**FIGURE 7:**
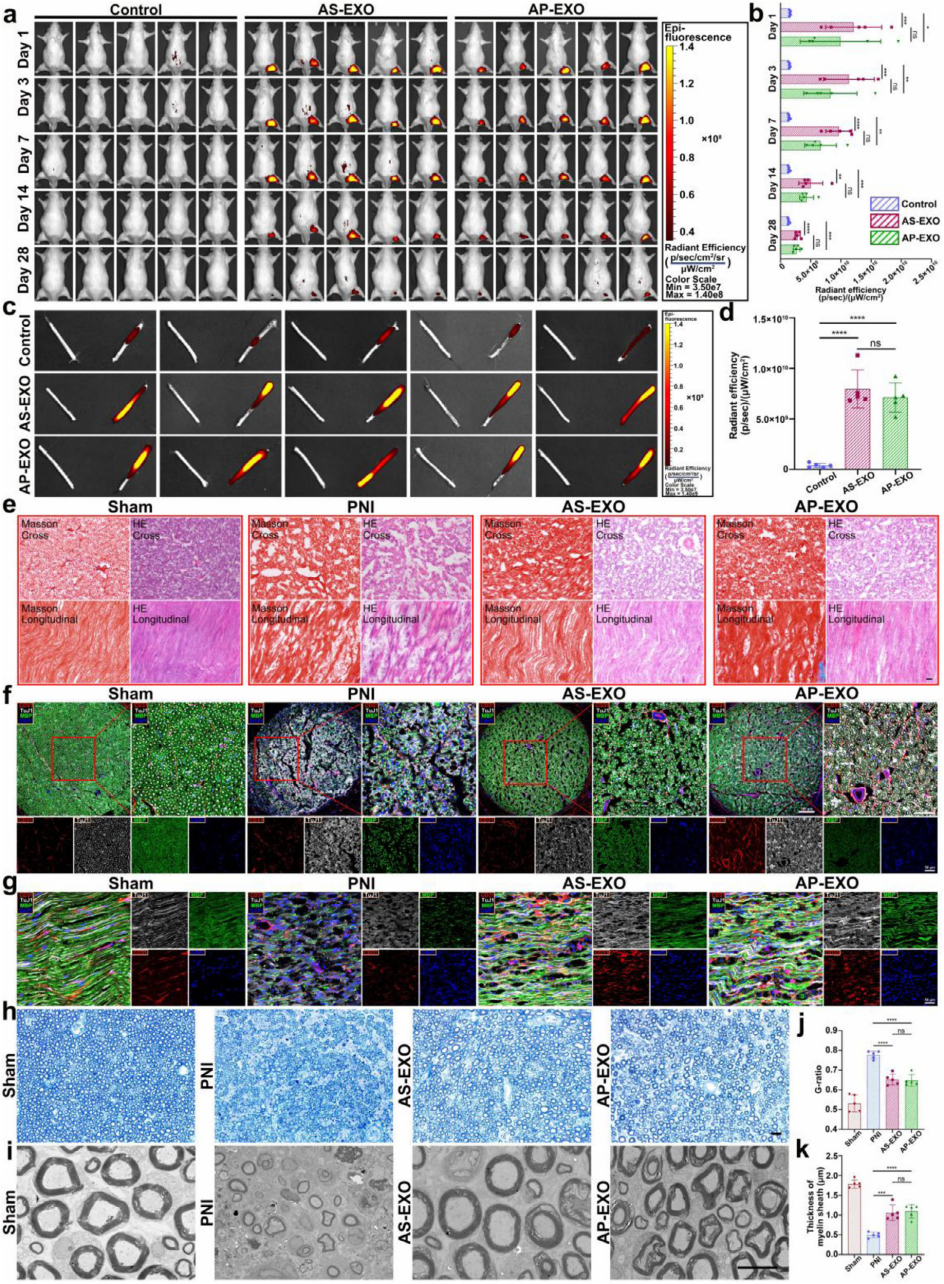
AB‐EXO facilitated angiogenesis and nerve repair. (a) Representative in vivo images for each group on days 1, 3, 7, 14, and 28 after surgery. (b) The radiant efficiency in different groups (*n* = 5). (c,d) Ex vivo fluorescence images of sciatic‐nerve specimens with corresponding statistical summary (*n* = 5). (e) Representative H&E‐ and Masson‐stained longitudinal and cross‐sections of sciatic nerves. Scale bar, 20 µm. (f) Representative quadruple immunofluorescence images (CD31/TuJ1/MBP/DAPI) of sciatic‐nerve cross‐sections. Scale bar, 100 and 50 µm. (g) Representative immunofluorescence images of longitudinal sciatic‐nerve sections. Scale bar, 50 µm. (h) Representative TB–stained sections of sciatic nerve. Scale bar, 20 µm. (i–k) Representative TEM images of sciatic‐nerve sections and corresponding quantification of G‐ratio and myelin sheath thickness (*n* = 5). Scale bar, 10, 2 and 1 µm. ns = not significant, *p < 0.05, **p < 0.01, ***p < 0.001, ****p < 0.0001.

To map the spatiotemporal distribution of AB‐EXO in vivo, DiR‐labeled AS‐EXO and AP‐EXO were micro‐injected beneath the sciatic epineurium, and their retention was tracked by in vivo fluorescence imaging on days 1, 3, 7, 14, and 28 post injection. In vivo imaging revealed that both AS‐EXO and AP‐EXO distributed broadly throughout the nerve and persisted for at least four weeks—far exceeding the retention observed in the control group—while the signal intensities of AS‐EXO and AP‐EXO remained indistinguishable at each time point examined (Figure 7a,b). Subsequent ex vivo bioluminescence of excised sciatic nerves confirmed comparable signal intensities between AS‐EXO and AP‐EXO, underscoring their equally robust affinity for neural tissue (Figure 7c,d). HE and Masson trichrome staining revealed that AS‐EXO‐ and AP‐EXO‐treated nerves exhibited well‐preserved architecture, and negligible vacuolation—striking improvements over the PNI group (Figure 7e). To visualize the pro‐regenerative effects of AB‐EXO, cross and longitudinal sciatic‐nerve sections were subjected to CD31, TuJ1, MBP, and DAPI quadruple immunofluorescence staining across different groups. Representative images revealed that both AS‐EXO and AP‐EXO potently augmented angiogenesis and accelerated axonal regrowth (Figure 7f,g). TB staining and TEM further demonstrated that AS‐EXO‐ and AP‐EXO‐treated nerves exhibited substantially thicker myelin sheaths and a markedly reduced G‐ratio compared with the PNI group, underscoring the potent remyelinating capacity of AB‐EXO (Figure 7h–k).

To substantiate the superior regenerative potential of rapid‐growth antler blood, we directly compared the therapeutic efficacy of AB‐EXO against BS‐EXO in our nerve injury model. Histological examination via H&E and Loyez staining revealed that nerves treated with AB‐EXO displayed substantially better architectural integrity and markedly reduced vacuolation compared with those receiving BS‐EXO (Figure S19). To further delineate the differential regenerative capacity between these two exosome populations, we performed quadruple immunofluorescence staining (CD31/TuJ1/MBP/DAPI) on both cross‐ and longitudinal sciatic nerve sections. The resultant images demonstrated that AB‐EXO elicited significantly more robust angiogenesis and accelerated axonal regrowth beyond the levels achieved by BS‐EXO (Figure S19). Moreover, to establish that IMP3+ECs are indispensable for PVN orchestration and nerve repair in vivo, we performed AAV‐mediated endothelial IMP3 manipulation across multiple treatment groups (AAV‐NC, AAV‐shIMP3, AAV‐IMP3, with or without AB‐EXO). Histological examination revealed that IMP3 knockdown largely abolished the morphological improvements conferred by AB‐EXO, as evidenced by disrupted nerve architecture and increased vacuolation (Figure S20). Quadruple immunofluorescence (CD31/TuJ1/MBP/DAPI) further showed that AB‐EXO–induced angiogenesis, axonal regrowth, and remyelination were profoundly suppressed upon IMP3 silencing, whereas IMP3 overexpression enhanced these regenerative hallmarks (Figure S20). These findings establish a causal requirement for endothelial IMP3 in AB‐EXO–mediated nerve regeneration, confirming that IMP3+ECs serve as essential mediators of the regenerative response.

To further substantiate the minimal immunogenicity of local AB‐EXO delivery in PNI treatment, we investigated the local immune landscape at the injury site via the analysis of macrophage M1/M2 polarization. The results showed no significant difference in M1 polarization between the PNI and AB‐EXO groups; however, M2 polarization was significantly increased in the AB‐EXO treatment groups (Figure S21). Furthermore, we examined the impact of AB‐EXO on hematological parameters, hepatic and renal function markers (ALT, AST, CR, BUN), and systemic inflammatory indicators (IL10, IL‐4, IL‐1β, and TNF‐α) in PNI models. Results revealed no significant abnormalities among the different treatment groups, suggesting that AB‐EXO administration had no detrimental effects on major physiological functions (Figure S21). Histological analysis of major organs (heart, liver, spleen, lung, and kidney) via H&E and Masson staining was also conducted to comprehensively assess the overall safety profile of AB‐EXO. Moreover, histological analysis of major organs revealed no significant tissue damage in cardiac muscle, liver, spleen, lung parenchyma, or renal glomeruli (Figure S21).

### 2.9 AB‐EXO Facilitated the Innervation of Injured Nerves

**FIGURE 8:**
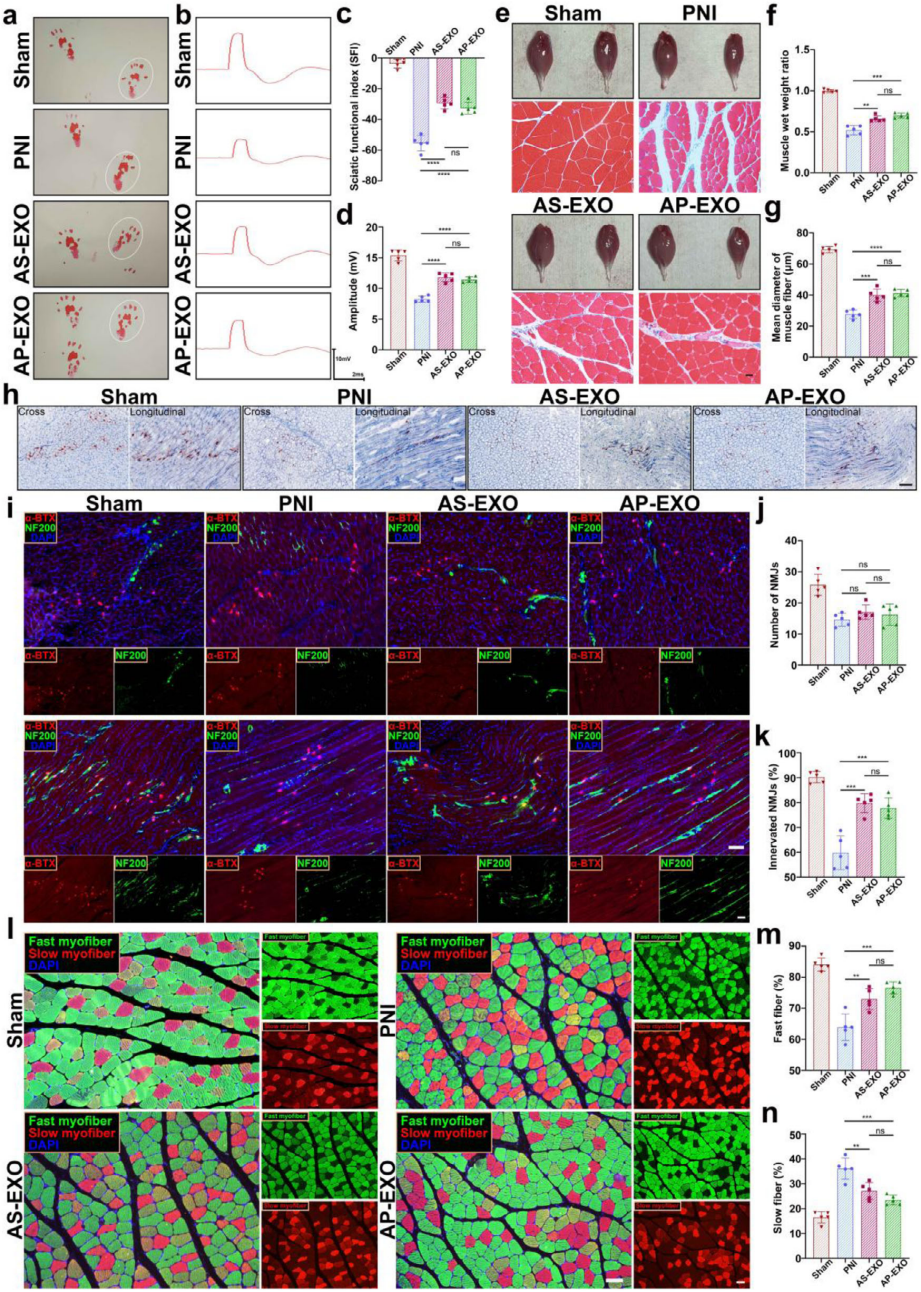
AB‐EXO enhanced reinnervation following PNI in vivo. (a,c) Representative ipsilateral and contralateral hind‐paw footprints for each group at 28 days post‐operation, with SFI quantification (*n* = 5). (b,d) Representative CMAP traces for each group at 28 days post‐operation, with statistical summary (*n* = 5). (e) Representative macroscopic images and Masson‐stained cross‐sections of gastrocnemius muscle from each group. Scale bar, 20 µm. (f,g) Quantification of the ipsilateral/contralateral weight ratio and the mean fiber diameter (*n* = 5). (h) Representative AChE–stained cross and longitudinal sections of gastrocnemius muscle. Scale bar, 200 µm. (i) Immunofluorescence staining of the NMJ of the gastrocnemius muscle. Scale bar, 100 µm. (j,k) Quantification of the NMJ numbers and the ratio of innervated NMJs (*n* = 5). (l) Representative immunofluorescence images of fast‐myosin and slow‐myosin in gastrocnemius‐muscle cross‐sections. Scale bar, 50 µm. (m,n) Quantification of the fast‐ and slow‐type fiber percentages in gastrocnemius muscle (*n* = 5). ns = not significant, **p < 0.01, ***p < 0.001, ****p < 0.0001.

To quantify motor recovery alongside nerve repair, walking‐track analysis was employed. Sciatic functional index (SFI) derived from footprint analysis revealed well‐spread, clearly defined prints in the sham, AS‐EXO, and AP‐EXO groups, whereas the PNI group displayed severe contractures. Correspondingly, SFI values for AS‐EXO and AP‐EXO were notably higher than those for PNI (Figure 8a,c). Electrophysiological recordings showed that both AS‐EXO and AP‐EXO markedly restored nerve conduction, as evidenced by substantially larger compound muscle action potential (CMAP) amplitudes at the injured side compared with the PNI group (Figure 8b,d). Gastrocnemius mass, a sensitive proxy for sciatic re‐innervation, was assessed by weighing bilateral muscles and calculating the injured‐to‐contralateral ratio. Muscles from AS‐EXO and AP‐EXO groups exhibited notably greater mass ratios than those from PNI animals, indicating marked attenuation of denervation‐induced atrophy. Moreover, Masson staining revealed that the mean muscle‐fiber diameter in both AS‐EXO and AP‐EXO groups was larger than in the PNI group, underscoring the preservation of muscle architecture following exosome therapy (Figure 8e–g). We examined the structure of the neuromuscular junctions (NMJs) using AChE staining and immunofluorescence staining, and found that PNI induced muscle denervation, as evidenced by a decrease in both the number of NMJs and the muscle innervation ratio. Notably, treatment with AB‐EXO significantly increased the rate of nerve ending innervation compared to the PNI group, while no significant changes were observed in the number of NMJs (Figure 8h–k). Following PNI, denervation preferentially targets fast‐twitch fibers; subsequent reinnervation by slow‐motor‐axon sprouts drives their phenotypic conversion toward a slow‐twitch identity [30]. Furthermore, AS‐EXO and AP‐EXO administration effectively reversed the PNI‐induced shift in fiber‐type composition, restoring a fast‐twitch dominant phenotype (Figure 8l–n). These results suggest that both AS‐EXO and AP‐EXO greatly accelerated neurologic functional recovery after nerve injury.

To validate the superior regenerative potential of rapid‐growth antler blood, we compared AB‐EXO with BS‐EXO. Histologically, AB‐EXO treatment preserved muscle architecture with significantly larger mean fiber diameters, while immunofluorescence revealed a higher rate of NMJ innervation relative to BS‐EXO (Figure S22). Furthermore, AB‐EXO effectively reversed the PNI‐induced fiber‐type shift, restoring a fast‐twitch dominant phenotype absent in BS‐EXO‐treated animals. Collectively, these results demonstrate that AB‐EXO accelerate functional recovery far more effectively than BS‐EXO after nerve injury (Figure S22). To establish that IMP3+ECs are indispensable for PVN orchestration and nerve repair in vivo, we evaluated functional recovery across AAV‐NC, AAV‐shIMP3, and AAV‐IMP3 cohorts with or without AB‐EXO treatment. Histological analysis revealed that the enhanced muscle‐fiber diameter and architectural preservation achieved with AB‐EXO treatment were substantially undermined following endothelial IMP3 silencing (Figure S23). Furthermore, the AB‐EXO‐induced reversal of PNI‐driven fiber‐type switching was nullified upon IMP3 knockdown, confirming that endothelial IMP3 expression is a prerequisite for AB‐EXO mediated functional and structural restoration (Figure S23).

## 3 Discussion

ECs within peripheral nerve were tracked by a recent lineage‐tracing scRNA‐seq study, unveiling heterogeneous subpopulations and dynamic fate transitions that span intact and regenerating peripheral nerves [9]. Single‐cell atlases of partially resected liver, spleen, and colon have resolved discrete angiogenic and proliferative EC clusters that are conspicuously absent from the brain or spinal cord [31]. This spatial restriction implies that a dormant yet recruitable mitotic EC reservoir underpins the heightened regenerative competence of peripheral nervous system (PNS). Scattered, FoxM1‐positive ECs—rare mitotic specialists that orchestrate vascular repair in other tissues—were likewise identified within the peripheral nerve vasculature [32]. Moreover, the repair angiogenic program re‐engineers the nerve's internal architecture, transmuting a default fibrotic scar into a permissive, axon‐bridging conduit that orchestrates regeneration. The organizational role of neo‐vessels in restructuring the nerve stroma critically depends on their topography and crosstalk with subsets of platelet‐derived growth factor receptor β^+^ mesenchymal cells (PDGFRB^+^ MSCs), possibly mediated by morphogenic signals expressed by angiogenic ECs [33, 34]. In central nervous system (CNS), stroke triggers subventricular zone (SVZ) progenitors to generate neurons that colonize a peri‐infarct neurovascular niche, where immature neurons intimately associate with remodeling vasculature [35]. Such a neurovascular niche defines the formation of a novel strong regenerative microenvironment and reveals the regenerative potential of ECs after CNS injury. Sparse, chaotic neovascularization at CNS lesions stalls scar remodeling, entrapping the wound in a chronically non‐resolving state [36, 37]. Hence, precisely orchestrated neovascularization is indispensable for reconfiguring the nascent, disorganized scar into a structured mesenchymal scaffold that channels regenerating nerve fibers in PNS. Repair angiogenesis transiently reactivated developmental vascular programs and unleashed an expanded cadre of tip cells that bore the migratory and extracellular matrix (ECM)–remodeling signatures characteristic of pathological angiogenesis [19, 38]. In nerve repair, the angiogenic program ignites rapidly yet is just as swiftly extinguished; by contrast, its chronic engagement foreshadows incomplete healing [39, 40]. These findings highlight the therapeutic imperative of recapitulating the precise spatiotemporal choreography of angiogenesis across discrete repair phases. ECs mobilized from injured peripheral nerves display intrinsically modest regenerative competence, constraining the angiogenic thrust needed for robust neural repair. Pinpointing, or bioengineering, more potent EC subsets could thus redefine the vascular contribution to peripheral‐nerve regeneration. State‐of‐the‐art scRNA‐seq has become the benchmark for disentangling vascular heterogeneity across the human brain, kidney, liver, and lung [41]. However, the application of scRNA‐seq to unraveling the vascular niche within peripheral nerve has been obfuscated due to the difficulty in obtaining adequate numbers of ECs from peripheral nerve. Thus, we sorted CD31^+^CD45^−^ ECs from peripheral nerves to generate comprehensive scRNA‐seq of EC molecular profiles within PNS. The EC atlas resolved four transcriptionally distinct clusters: angiogenic ECs (AECs), neuro‐inflammatory ECs (NIECs), barrier ECs (BECs) and lymphatic ECs (LECs). Among these, AECs surge in number post injury and ignite the angiogenic wave that underpins effective nerve regeneration. This injury‐triggered expansion reveals an underlying molecular–functional heterogeneity within the nerve vasculature, reprogramming the endothelial landscape into a distinctly pro‐regenerative configuration. Notably, AECs express core angiogenesis genes, including APLNR, FABP4, PLVAP and CXCR4 [42, 43, 44]. The combined expression of unique gene profiles, including GPIHBP1, RBP7, CXCL12, and COL4A1 characterizes AECs [18, 45, 46, 47]. A rich repertoire of AEC‐specific signature combinations is available to isolate these specialized vascular cells or to direct precise genetic manipulation in future work.

Annual antler regeneration—unique among mammalian appendages—provides an unparalleled natural model for dissecting the mechanisms of mammalian organ renewal. This regeneration process relies on both local cues—most critically antler stem cells (AnSCs) resident in the pedicle periosteum (PP) or in the early antler blastema —and systemic signals delivered by circulating blood [16, 48]. AnSCs constitute a promising, translation‐ready source for cutaneous repair. The population comprises 1) antlerogenic periosteal cells that initiate pedicle and first‐antler formation, 2) pedicle periosteal cells that drive annual renewal, and 3) reserve mesenchymal cells that fuel rapid outgrowth [49]. In AnSC–treated wounds, VEGF, FGF1, and HGF were all sharply elevated, likely orchestrating the switch to a regenerative, rather than merely reparative, healing program [50]. A recent study identified an unprecedented population of MSCs—antler blastema progenitor cells (ABPCs)—that alone can initiate de novo antler generation [12]. Unlike conventional MSCs—whether derived from bone marrow, adipose, or umbilical cord—that senesce after 10–15 passages, ABPCs retain vigorous proliferation and full regenerative potency beyond 50 passages [51, 52]. Furthermore, antler expansion is paralleled by explosive angiogenesis and neurogenesis, driven by the dense array of chemotactic signals released by ABPCs [12]. For effective tissue repair, MSCs rely on a triad of competencies—potent differentiation, immune regulation, and secretory activity—with the paracrine pathway recognized as the focal point [53, 54]. Through a potent, multifactorial secretome, MSCs re‐engineer their niche—spurring new vessel growth, awakening local progenitors, guiding cellular trafficking and fate decisions, and sustaining viability and metabolic homeostasis [55]. Compared to canonical MSCs, AnSCs remain confined to the PP and are excluded from the overlying dermis, potentially endowing them with a uniquely potent paracrine arsenal [56]. Besides offering a more efficacious therapy for regeneration, the application of AnSC paracrine factors would be advantageous for the development of novel pro‐regenerative reagents including AnSC‐conditioned medium and AnSC‐EXO [57, 58]. Employing AnSC‐EXO in the clinic would replace living‐cell therapies with a standardized, cell‐free platform that eliminates donor‐to‐donor variability, bypasses concern over cell origin and immune‐compatibility, and enables exact dosing of purified paracrine cargo. This strategy promises safer, more effective regenerative agents whose outcomes are both predictable and precisely tunable [16]. Strikingly, excising the PP—the niche harboring AnSCs—does not impede regenerative cutaneous repair, indicating that systemic factors from the circulating blood must be involved in this generic regenerative wound healing [59]. Circulating factors within antler blood orchestrate generic wound repair by tipping the balance toward anti‐inflammation, restraining pro‐inflammatory signaling, driving robust angiogenesis, blocking myofibroblast conversion, elevating the TGF‐β3/β1 ratio, and directing orderly collagen remodeling [11]. Collectively, these findings reveal that while full antler regeneration demands both local AnSC‐derived cues and systemic factors within circulating blood, the latter alone can establish a pro‐regenerative niche for tissue repair.

Antler blood‐derived EXO (AB‐EXO) retain the full regenerative signaling networks of the native tissue microenvironment, offering a more faithful representation of in‐vivo conditions than AnSC‐EXO derived from cultured antler stem cells and, consequently, yielding more comprehensive biological insights. In this study, we performed a combination of high‐throughput detection methods to evaluate the source of AB‐EXO [20]. Employing these innovative profiling techniques, we traced AB‐EXO to a hierarchical cellular origin: the majority arise from mesenchymal cells, ECs and immune populations, with secondary contributions from osteoblasts, pericytes and fibroblasts within the antler tissue. We also found that mesenchymal cells display the highest EXO‐secretory capacity, followed—at markedly lower levels—by ECs and then by all other resident cell types. The difference in EXO releasing ability among different antler cell types confirms that EXO are functional vesicles released from cells in a precisely controlled fashion. To uncover the presumptive regenerative drivers uniquely enriched in AB‐EXO, we subjected the vesicles to quantitative proteomic profiling. Quantitative profiling revealed pronounced enrichment of regeneration‐associated proteins—POSTN, IGFBP4, LCP1, TNC, LUM among others—collectively establishing a pro‐regenerative milieu within AB‐EXO [60, 61, 62, 63]. The robust enrichment of POSTN and LUM in AB‐EXO mirrors their striking up‐regulation in antler mesenchymal cells during active regeneration, where their expression escalates in lockstep with the antler's elongation rate [12]. Antler regeneration is driven by the precisely timed convergence of angiogenesis, neurogenesis, and immune modulation—a triad whose exquisite vascular component urges us to elucidate how AB‐EXO choreograph vascularization for flawless tissue repair [64]. Our study identified antler blood as the viable source of EXO and revealed the remarkable ability of AB‐EXO to enhance angiogenesis, which might be due to the unique pro‐angiogenic factors within the vesicles. To pinpoint how AB‐EXO imprint a pro‐angiogenic identity on ECs, we performed comprehensive proteomic profiling of ECs treated with AB‐EXO, quantifying the full spectrum of differentially expressed proteins. Strikingly, AB‐EXO reprogrammed ECs into an angiogenic state by transferring antler‐specific cues. Among these, IMP3—robustly up‐regulated in AB‐EXO treated ECs—emerged as the pivotal driver of this angiogenic conversion. IMP3 assembles into dynamic ribonucleoprotein complexes that orchestrate pre‐rRNA processing, ensuring the precise and efficient cleavage reactions essential for mature rRNA biogenesis [65, 66]. Furthermore, RRP9—a specialized module within the IMP3 complex—was found to engage the AKT signaling axis and physically associate with JUN, thereby coupling ribosomal biogenesis to pro‐angiogenic transcriptional programs [26]. Additionally, IMP3‐directed ribosome biogenesis constitutes a critical determinant of stem‐cell identity and functional competence [22]. Thus, IMP3 is considered to emerge as a pivotal orchestrator of tissue repair, governing both angiogenic expansion and neural regeneration. In our study, markedly elevated IMP3 expression was detected in ECs treated with AB‐EXO and was correlated with the induction of angiogenic phenotype. By systematically silencing or overexpressing IMP3, we delineated its dual role in driving angiogenesis and peripheral‐nerve regeneration. Collectively, our data position IMP3 as a promising therapeutic target for vascular and nerve repair. Pinpointing the pivotal factors within AB‐EXO would transform this abundant natural resource into an essentially limitless, clinically deployable therapeutic. Subsequent enrichment of AB‐EXO for these key effectors is poised to markedly amplify their pro‐regenerative potency across the spectrum of nerve repair. Additionally, AB‐EXO prompted us to spotlight IMP3 as a master pro‐angiogenic cue, guiding the discovery of a subset of ECs endowed with heightened regenerative capacity and thereby redefining the contribution of ECs to peripheral‐nerve repair. By mapping IMP3 expression onto our single‐cell atlas, we resolved a discrete population of IMP3‐high angiogenic endothelial cells—IMP3+AECs—that emerge as the dominant vascular architects of peripheral‐nerve repair.

While our work delivers a comprehensive molecular map of peripheral‐nerve ECs, its scope remains bounded by several caveats that merit attention. First, the scarcity of ECs within sciatic nerve tissue renders their isolation labor‐intensive and inherently low‐yield. Sample‐handling variables—dissociation kinetics, time‐to‐lysis, and any ex‐vivo culture interval—can rapidly remodel endothelial transcriptional states; therefore, maximizing EC yield while minimizing processing duration is critical to preserving in‐vivo signatures and ensuring that downstream findings are broadly generalizable. A further constraint is the risk that contaminating transcripts—derived from residual non‐ECs or introduced during tissue dissociation—can obscure or artefactually amplify the authentic gene‐expression signatures of ECs, thereby complicating downstream interpretation of the scRNA‐seq data. Despite the breadth of our analytical toolkit, the intrinsic sparsity of weakly transcribed genes and the phenotypic ambiguity of certain cell populations continue to elude precise detection. To move from signature to mechanism, future work should first leverage next‐generation cell‐sorting platforms—such as index‐sorting coupled with multiplexed surface‐marker panels—to achieve high‐purity isolation of angiogenic ECs. Subsequent in vitro gain‐ and loss‐of‐function perturbations, mapped back to the transcriptomic atlas generated here, will then be required to causally validate the predicted signaling axes and quantify their dysregulation in vascular disease. Here, we demonstrated that EXO isolated from antler plasma (AP‐EXO) potently couple angiogenic activation with accelerated peripheral‐nerve regeneration. Besides, recent studies found that platelet‐rich plasma derived EXO (PRP‐EXO) have great potential in the field of tissue repair and regeneration, with anti‐inflammatory effects and positive regulation of cell biological activity [67, 68]. A parallel study revealed that PRP‐EXO dismantle neutrophil extracellular traps (NETs) via selective down‐regulation of MMP‐8, thereby reinstating a pro‐healing microenvironment and markedly accelerating diabetic wound closure [69]. These findings position antler PRP‐EXO as a potent, yet currently unexploited, therapeutic modality for peripheral‐nerve regeneration. While exosome administration has shown an acceptable safety window in both rodent and non‐human primate models, only extended surveillance can definitively rule out latent, dose‐dependent oncogenesis. Pinpointing the exclusive regenerative cargo within AB‐EXO—and subsequently transplanting these factors into scalable, non‐antler EXO—could transform ordinary sources into next‐generation therapeutics with superior repair capacity.

Collectively, our findings demonstrate the safety of local AB‐EXO administration in the rat PNI model. Nevertheless, the possibility of adverse immunological reactions warrants consideration for heterologous exosome therapies, although no such effects were detected herein. The rapidly expanding field of EV therapeutics and drug delivery systems necessitates a thorough understanding of potential immunogenicity, which is critical for developing safe and effective clinical products. Despite promising safety profiles in preclinical and early clinical studies, EV immunological clearance remains understudied, particularly in large animal models. Critical factors influencing EV immunogenicity include source, size, surface composition, internal cargo, manufacturing protocols, dosage, infusion rate, and biomolecular corona characteristics [70]. Hao et al. reported that antler blastema progenitor cell‐derived extracellular vesicles (ABPC‐EVs) offer a geroprotective approach for mitigating cellular senescence and reversing aging‐associated pathologies in skeletal and organ systems across rodent and nonhuman primate (NHP) models [71]. Upon intravenous delivery to aged rhesus macaques, ABPC‐EVs were found to traverse the blood–brain barrier efficiently, underscoring their promise for ameliorating brain aging. Moreover, the investigators established the favorable safety profile and minimal immunogenicity of ABPC‐EVs across rodent and NHP models. Such findings hold significant translational potential for the development of therapies targeting age‐related diseases. From a sourcing perspective, blood represents a high‐quality option for exosome isolation. Compared with stem cell‐derived exosomes, blood‐derived exosomes are more abundant and less immunogenic, thereby minimizing the risk of immune rejection. Additionally, blood collection is convenient and enables stable exosome production under safe conditions, providing a solid foundation for subsequent research and clinical applications [72]. Recent evidence has begun to shed preliminary insights into the role of blood derived EVs in many diseases. Chen et al. demonstrated that EVs isolated from the plasma of young mouse exhibit a clear cognitive‐enhancing ability in aged mice by rescuing mitochondrial dysfunction [73].

## 4 Conclusions

In summary, we present the single‐cell atlas of peripheral‐nerve‐specific ECs, revealing that injury has ignited a rapid surge of AECs that choreograph peripheral‐nerve revascularization. Mimicking nature's most efficient repair engine—the cyclically regenerating deer antler—we show that AB‐EXO recapitulate this program, delivering a multimodal pro‐regenerative cargo that accelerates both angiogenesis and axonal regrowth. AB‐EXO achieve this by unleashing IMP3, licensing a discrete IMP3+AEC subset to become the dominant vascular architects of neural repair. Thus, IMP3+AECs constitute the core effector population, and AB‐EXO serve as an IMP3‐targeted biologic that can be deployed to mobilize these cells for therapeutic nerve regeneration.

## 5 Experimental Section

### 5.1 Animals

Adult male Sprague–Dawley rats (12 weeks old, 300–400 g; Beijing Vital River Laboratory Animal Technology) were pair‐housed in individually ventilated cages under specific‐pathogen‐free (SPF) conditions (22 ± 1°C, 55 ± 5% humidity, 12 h light / 12 h dark cycle). Standard irradiated chow and reverse‐osmosis water were provided ad libitum. All experimental protocols were approved by the Animal Care and Use Committee of The First Affiliated Hospital of Zhengzhou University (Approval No. 2024‐KY‐2093) and conducted in accordance with the NIH Guide for the Care and Use of Laboratory Animals and the ARRIVE 2.0 guidelines.

### 5.2 Sciatic Nerve Injury

Following brief ether induction, anesthesia was maintained with intraperitoneal 2% sodium pentobarbital at 2 mL kg^−1^. The right sciatic nerve was exposed through blunt dissection along the gluteal muscle plane. At 5 mm distal to the sciatic notch, the nerve was crushed with Dumont #5 forceps for three cycles (10 s compression and 10 s rest each). Then a 2‐mm translucent band appeared at the lesion site. Sham‐operated animals underwent identical procedures except that the sciatic nerve was not crushed.

### 5.3 Preparation of Single‐Cell Suspensions

Sciatic nerves were rapidly excised from sham‐operated and injured rats (injury site ± 5 mm proximal and distal segments) and immediately immersed in ice‐cold tissue‐storage solution (Miltenyi Biotec). After mincing with fine scissors, nerve fragments were enzymatically dissociated in 1 mL PBS containing 1 mg/mL collagenase IV (Sigma) and 100 µg mL^−1^ DNase I (Sigma) in a 15 mL conical tube at 37°C for 30–45 min, with gentle agitation every 5 min and mechanical trituration (20 strokes with a 1000 µL pipette) every 10 min. The resulting cell suspension was filtered through a 40 µm cell strainer (BD), stained with anti‐CD45 antibody (Miltenyi Biotec) and anti‐CD31 antibody (Miltenyi Biotec), and CD45^−^CD31^+^ cells were sorted on a BD FACSAria Fusion. Myelin debris was removed using myelin‐removal beads (Miltenyi), and cells were resuspended in HBSS (Sigma) containing 0.04% BSA (Miltenyi). Viability (> 90%) was verified by propidium iodide exclusion and hemocytometry. Single‐cell RNA‐seq libraries were generated on a 10× Chromium Controller (v3 chemistry, 10× Genomics) and sequenced on an Illumina HiSeq 4000 (paired‐end 150 bp). Cell barcodes and UMIs were processed with Cell Ranger.

### 5.4 ScRNA‐Seq Data Analysis

Single‐cell RNA‐seq (scRNA‐seq) data were processed with Cell Ranger (10× Genomics) for read alignment, barcode demultiplexing, and UMI quantification. Single‐cell encapsulation, barcoding and cDNA amplification were performed using the Chromium Single Cell 3′ v2 Reagent Kit according to the manufacturer's protocol. Filtered feature‐barcode matrices were imported into Seurat v3.2.3 and converted to sparse matrices. Cells with <200 detected genes or >20% mitochondrial reads were excluded. Highly variable genes (HVGs) were identified with FindVariableFeatures (selection.method = “vst”, nfeatures = 2000). Principal‐component analysis (PCA) was performed with SeuratRunPCA; the top 20 PCs were used for downstream analyses. Clustering (resolution = 0.6) was carried out with FindNeighbors followed by FindClusters; dimensionality reduction employed RunTSNE and RunUMAP (perplexity = 30 for t‐SNE). Cluster marker genes were visualized with VlnPlot. Functional enrichment of gene lists was performed using clusterProfiler::enricher (Benjamini–Hochberg FDR < 0.05). Clusters were identified with Seurat's FindClusters (resolution = 0.8). Marker genes for each cluster were defined as the top 20 differentially expressed genes ranked by average log2 fold change. Dimensionality reduction and visualization employed RunUMAP; the resulting Uniform Manifold Approximation and Projection (UMAP) embeddings are displayed in all figures. Cluster‐specific genes were identified with FindAllMarkers (min.pct = 0.25, logfc.threshold = 0.25). Differential expression between a given cluster and all remaining cells was assessed via the Wilcoxon rank‐sum test implemented in FindMarkers (default parameters).

### 5.5 Cell Trajectory Analysis

RNA velocity and pseudotime analyses were performed with scVelo (v0.2.3) and Monocle2 (v2.18.0), respectively. Velocyto (v0.17.17) converted aligned BAMs into loom files of spliced/unspliced counts; scVelo normalized the top 4 000 variable genes, computed moments on 20 Seurat PCs, estimated gene‐specific velocities with the stochastic model, and projected the velocity graph onto the UMAP embedding. For pseudotime reconstruction, integrated intact, 3 dpc and 7 dpc EC datasets were imported into Monocle2; unsupervised DDRTree ordering used Seurat‐derived variable genes, the root was set to the mitotic cluster identified by GM_state, and trajectories were visualized with plot_cell_trajectory, plot_genes_branched_pseudotime and plot_genes_branched_heatmap.

### 5.6 Cell‐Cell Communication Prediction

To dissect intercellular communication at single‐cell resolution, we employed CellChat v1.1.3, which curates 60% paracrine/autocrine, 21% ECM‐receptor, and 19% cell–cell contact signaling pathways [74]. Interaction strength was quantified from differentially expressed ligand–receptor pairs, enabling systematic comparison across predefined cell groups.

To model how intercellular signals shape downstream transcriptional programs, we applied NicheNet. The algorithm integrates prior ligand–receptor interaction data, signal‐response compendia, and gene‐regulatory networks to predict, for each ligand expressed by sender cells, its most probable target genes within interacting receiver cells [23]. We set the IMP3+AECs as the sender cell, other ECs (including IMP3‐AECs, NIECs, BECs and LECs) as the receiver cells, and calculated the differential expression of receiver cells and input it as a target gene set. Then NicheNet was used to calculate how the IMP3+AECs regulate and act on observed differential genes.

### 5.7 Exosomes Isolation and Characterization

Antler blood was presented by Prof. Piao from Yanbian University Hospital (Animal Ethics Approval No. 2024346). Antler blood was harvested from male sika deer (*Cervus nippon*) during casting events in the rapid‐growth season (June–August). To isolate exosomes from antler‐derived fluids, we collected serum and plasma immediately after natural casting of the deer antlers. Antler serum was harvested by collecting whole blood directly from the antler vein into sterile, additive‐free tubes. After clot formation at 4°C for 3–4 h, the pale‐yellow serum was gently aspirated and aliquoted into sterile cryovials for downstream use. For antler plasma, whole blood was drawn directly into EDTA tubes, gently inverted to mix, and processed within 1 h. After an initial centrifugation (1600 × g, 4°C, 10 min), the supernatant was transferred to fresh 1.5 mL tubes and re‐centrifuged (16 000 × g, 4°C, 10 min). The clarified plasma was carefully aspirated and aliquoted into sterile cryovials for downstream applications.

Antler serum and plasma were first cleared of cells and debris by sequential centrifugation (800 × g, 10 min; 5000 × g, 10 min). The resulting supernatants were passed through a 0.22 µm filter (Millipore) and then ultracentrifuged at 100 000 × g for 2 h at 4°C (Micro Ultracentrifuge, Himac, Japan) to pellet EXO. The EXO derived from antler serum (AS‐EXO) and antler plasma (AP‐EXO) were resuspended in PBS, and their protein content was quantified with a BCA Protein Content Assay Kit (Boxbio, China). For the cell and animal treatment, 100 µg mL^−1^ EXO were used.

EXO were fixed in 2% glutaraldehyde. A 5‐µL aliquot of the suspension was applied to a carbon‐coated copper grid for 5 min, negatively stained with 2% phosphotungstic acid for another 5 min at room temperature, and then examined by transmission electron microscopy (TEM, HITACHI, Japan) to visualize morphology. Size distribution and zeta potential were determined by nanoparticle tracking analysis (NTA) using a NICOMP Nano‐ZLS Z3000 instrument (Beckman Coulter, USA). The exosomal markers CD63 (1:1 000, Abcam), CD81 (1:1 000, Abcam), TSG101 (1:1 000, Zenbio) and Calnexin (1:1 000, Abcam) were detected by Western blot.

### 5.8 Integrated ScRNA‐Seq Profiling of Antler with Proteomic Mapping of AB‐EXO

We downloaded scRNA‐seq data from NCBI database (accession ID: PRJNA931044), providing scRNA‐seq data of antler cells [12]. Single‐cell RNA‐seq data from antler tissue collected at 5 days after casting (5 dac) were analyzed with Seurat v3.0 in R. Highly variable genes were identified per sample using FindVariableFeatures. Integration anchors between antler datasets were discovered with FindIntegrationAnchors, followed by data integration via IntegrateData. Standard Seurat workflows were then applied: cells were clustered with FindClusters and visualized in two dimensions using RunTSNE. Cluster‐specific marker genes—defined as transcripts exhibiting notably higher expression within one cluster versus all others—were identified with Seurat's FindAllMarkers function. The resulting gene lists were then intersected with the AB‐EXO proteome to pinpoint proteins shared between each antler cell cluster and the EXO cargo. The expression levels of the genes which encode the selected AB‐EXO proteins among the different antler cell clusters were then illustrated by the “DoHeatmap” function in the “Seurat” package. In order to evaluate the ability of single cell or single cluster of cells to secrete EXO, we calculated this using the method described in a previous study [20]. Then we calculated the capacity of EXO secretion of antler cells. The EXO proteomic was supported by Shanghai OE Biotech CO.

### 5.9 EXO Fluorescence Labelling In Vitro

AS‐EXO and AP‐EXO were fluorescently labeled by incubation with 1,1′‐dioctadecyl‐3,3,3′,3′‐tetramethylindocarbocyanine perchlorate (DiI; FD‐CDI001, Biolight) at 10 mg mL^−1^ (dye/EXO volume ratio 1:100) for 30 min at 37°C in the dark. Unbound dye was removed by dialysis against PBS for 12 h at 4°C using a 100 kDa molecular‐weight‐cutoff membrane (Shanghai Yuanye Bio‐Technology). Cells seeded in 20 mm glass‐bottom dishes (Nest, China) were incubated with DiI‐labeled EXO for 2, 6, 12, or 24 h, counterstained with DAPI, and imaged by confocal laser‐scanning microscopy.

### 5.10 Cell Cycle

Cells were harvested from 6‐well plates, washed with PBS, and fixed. Cell‐cycle distribution was determined using the Cell Cycle Staining Kit (KeyGEN BioTECH, China) following the manufacturer's instructions and analyzed by flow cytometry.

### 5.11 Western Blot Analysis

Cells were lysed in RIPA buffer (Epizyme, China) supplemented with Phenylmethylsulfonyl Fluoride (PMSF; SJ‐MX2470, Sparkjade). Protein concentration was determined by BCA Protein Content Assay Kit. Equal amounts of protein were resolved on PAGE gels according to the PAGE Gel One‐Step Preparation Kit (Swiss Affinibody LifeScience AG, China) and transferred to PVDF membranes (Millipore, USA). After blocking with Protein‐Free Rapid Blocking Buffer (Epizyme, 1 h, room temperature), membranes were incubated overnight at 4°C with the following primary antibodies: IMP3 (1:1 000, Thermo Fisher), AKT (1:10 000, Abcam), p‐AKT (1:5 000, Abcam), ERK1/2 (1:1 000, CST), p‐ERK1/2 (1:2 000, CST), PLVAP (1:1 000, Proteintech), CDK1 (1:1 000, AntibodySystem), CCND1 (1:1 000, Abcam), and GAPDH (1:1 000, K200057M, Solarbio). Membranes were washed, incubated with HRP‐conjugated secondary antibodies (Solarbio), and visualized using the Amersham Imager 600. GAPDH served as the loading control.

### 5.12 Proteomic Analysis

4D label‐free quantitative (LFQ) proteomics was performed by Jingjie PTM BioLabs (Hangzhou, China). EC pellets were lysed mechanically, clarified by centrifugation, and protein concentrations determined by BCA assay. Proteins were precipitated with trichloroacetic acid, washed with ice‐cold acetone, re‐dissolved in triethylammonium bicarbonate, and digested overnight with trypsin. Peptides were desalted on a Strata‐X SPE cartridge, separated on a NanoElute UHPLC system, and subjected to on‐line capillary ionization and data‐independent acquisition on a timsTOF Pro mass spectrometer. A reverse‐decoy database was searched to control false‐discovery rate (FDR ≤ 1%), and common contaminants were removed.

Data quality was assessed by Pearson correlation and principal‐component analysis (PCA). Differentially expressed proteins were identified by two‐tailed t‐tests; proteins with an LFQ fold‐change > 1.2 (or < 0.83) and a *p*‐value < 0.05 were considered up‐ or down‐regulated, respectively. Gene Ontology (GO) enrichment analysis was performed with the GO Term database, while KEGG and Reactome databases were used for pathway annotation. Protein–protein interactions (PPIs) were queried in STRING v11.0 and visualized with the networkD3 R package.

### 5.13 Construction of LV‐IMP3, LV‐shIMP3 and LV‐NC ECs

ECs stably overexpressing IMP3 (LV‐IMP3), knocked down for IMP3 (LV‐shIMP3), or carrying a non‐targeting control (LV‐NC) were generated with lentiviral vectors (Genechem, China). Briefly, 1 × 10^6^ cells per well were seeded in 6‐well plates and cultured overnight, then transduced with lentivirus according to the manufacturer's protocol. Stable lines were selected with G418 (Beyotime, China).

### 5.14 RT‐qPCR Analysis

Total RNA was isolated with RNAiso Plus (Takara, Japan) and reverse‐transcribed using Evo M‐MLV RT Kit (ACCURATE BIOTECHNOLOGY, China) according to the manufacturer's protocol. Quantitative PCR was performed in 20 µL reactions containing cDNA, gene‐specific primers, and NovoStart SYBR qPCR SuperMix Plus (Novoprotein Scientific, China) on a real‐time PCR system. We set three replicates in each group and used the 2^ΔΔCT^ method. The primer sequences used for qPCR were as follows:

IMP3‐forward: 5’‐TGTATGCTCTCGGCTTGGTG‐3’,

IMP3‐reverse: 5’‐TCGCGCTCCTCATTGTACTC‐3’;

GAPDH‐forward:5’‐CAGGAGGCATTGCTGATGAT‐3’,

GAPDH‐reverse: 5’‐GAAGGCTGGGGCTCATTT‐3’.

### 5.15 AAV Injection In Vivo

Endothelial‐cell (EC)‐specific adeno‐associated viral (AAV) vectors were engineered with IMP3 expression placed under the control of the endothelial TIE promoter, thereby restricting transgene activity to ECs within peripheral nerves (Genechem, China). Under brief isoflurane anesthesia, rats received a single 10‐µL epineural microinjection of AAV‐NC (control), AAV‐IMP3 (overexpression), or AAV‐shIMP3 (knockdown) into the sciatic nerve. Subsequent experiments were performed at 14 days post injection.

### 5.16 Flow Cytometry Analysis of Sciatic Nerves

Sciatic nerves were harvested from AAV‐NC, AAV‐IMP3, and AAV‐shIMP3 rats at 7, 14, and 28 days post‐injury. After three washes with HBSS, nerves were minced and digested in tissue‐dissociation solution (Reward, China) for 1 h at 37°C. Then cells were resuspended in PBS buffer containing 1%–2% fetal bovine serum (KX‐A1233, Inner Mongolia Wanrui Biotechnology Co.,Ltd). The resulting cell suspension was passed through a 40 µm strainer and pelleted at 300 × g, 4°C for 5 min. Cells were resuspended in 1× Flow Cytometry Staining Buffer (R&D Systems) and stained with PE‐Cyanine7‐conjugated anti‐CD31 (1:50, Thermo Fisher) and APC‐conjugated anti‐Ki67 (1:50, Thermo Fisher). Following washes, samples were acquired on a BD FACSCanto II using FACSDiva v7 software and analyzed with FlowJo v10.8.

### 5.17 Multicolor Immunofluorescence Staining

Sciatic nerves were fixed in 4% paraformaldehyde (PFA) and sectioned in both cross and longitudinal orientations. Tissue sections were immunostained with rabbit anti‐CD31 (1:100, Abcam), chicken anti‐MBP (1:100, Invitrogen), and mouse anti‐TuJ1 (1:100, Abcam) primary antibodies, followed by Alexa Fluor 555‐conjugated donkey anti‐rabbit (1:200, Abcam), Alexa Fluor 594‐conjugated donkey anti‐chicken (1:200, Thermo Fisher), and Alexa Fluor 647‐conjugated donkey anti‐mouse (1:200, Abcam) secondary antibodies. For the investigation of macrophage polarization in injured sites, nerves were immunostained with chicken anti‐CD68 (1:100, Abcam), mouse anti‐iNOS (1:100, Abcam), and rabbit anti‐ARG1 (1:100, Abcam) primary antibodies, followed by Alexa Fluor 647‐conjugated donkey anti‐rabbit (1:200, Abcam), Alexa Fluor 488‐conjugated donkey anti‐chicken (1:200, Thermo Fisher), and Alexa Fluor 555‐conjugated donkey anti‐mouse (1:200, Abcam) secondary antibodies. Images were acquired on a confocal laser‐scanning microscope.

### 5.18 TB Staining and TEM Assessments

The sciatic nerves were harvested and put in 2.5% glutaraldehyde overnight. Then the tissues were immersed in 1% osmic acid for 2 h and dehydrated with acetone. The samples were encapsulated in epoxy resin and oven‐dried. The tissues were sectioned into 0.5 µm semi‐thin cross‐sections and 70.0 nm ultrathin cross‐sections. The semi‐thin sections were stained with toluidine blue (TB) and observed using a light microscope. The ultrathin cross‐sections were examined using the TEM. ImageJ software was used to measure myelin sheath thickness and G‐ratio (inner axonal diameter to fiber diameter ratio).

### 5.19 Bioluminescence Imaging

To track AS‐EXO and AP‐EXO in vivo, vesicles were labeled with the lipophilic dye DiR (Umibio, China) per the manufacturer's protocol, then dialyzed (100 kDa MWCO, 12 h, 4°C) to remove unincorporated dye; PBS processed identically served as a control. A single 10 µL epineural injection of DiR‐labeled EXO (1 µg EXO per rat) was delivered to the sciatic nerve with a Hamilton micro‐syringe. In vivo imaging was performed with an IVIS Spectrum (PerkinElmer) at 1, 3, 7, 14, and 28 days post‐injection. At day 28, sciatic nerves were harvested for high‐resolution fluorescence imaging to confirm EXO localization.

### 5.20 Electrophysiological Assessment

The electrophysiological assessment was conducted using previously developed methods [75]. Twenty‐eight days post‐operation, sciatic nerve function was assessed with the MD3000‐C multichannel physiological recording system (Anhui Zhenghua Biological Instrument, China) [76]. Under anesthesia, the sciatic nerve was exposed and bipolar stimulating electrodes were positioned immediately proximal to the crush site; a recording electrode was simultaneously inserted into the ipsilateral gastrocnemius. Compound muscle action potentials (CMAPs) were evoked by single electrical pulses and recorded for inter‐group comparison.

### 5.21 Histological and Immunofluorescence Staining of Muscle

At 28 days post‐operation, bilateral gastrocnemius muscles were immediately excised and weighed to determine the relative wet‐weight ratio (ipsilateral/contralateral). The experimental muscle belly was fixed in 4% PFA, paraffin‐embedded, and sectioned. Masson staining was performed to assess fibrosis; images were acquired on a light microscope and mean fiber diameter was quantified with ImageJ. Adjacent sections were immunolabeled with rabbit anti‐MYH1 (1:100, Servicebio) and mouse anti‐MYH7 (1:100, Servicebio), followed by Alexa Fluor 488 donkey anti‐rabbit (1:200, Abcam) and Alexa Fluor 594 donkey anti‐mouse (1:200, Thermo Fisher), and imaged by fluorescence microscopy.

### 5.22 Statistical Analysis

Statistical analysis was conducted using GraphPad Prism 8.0 (GraphPad Software, La Jolla, CA, USA). The results were presented as mean ± SD. One‐way ANOVA was used for comparisons within multiple groups, and a two tailed unpaired Student's test was used for comparisons between two groups. *P* values < 0.05 were considered statistically significant.

## Author Contributions

Conceptualization: **J.H**., **M.P**., **Y.W**., and **N.Z**. Methodology: **J.H**., **Q.C**., and **A.J**. Investigation: **J.H**., **Q.C**., **A.J**., **X.Y**., and **H.Z**. Visualization: **J.H**., **W.G**., **J.C**., **J.D**., and **M.W**. Supervision: **M.P**., **Y.W**., and **N.Z**. Funding acquisition: **M.P**., **Y.W**., and **N.Z**. Project administration: **M.P**., **Y.W**., and **N.Z**. Writing – original draft: **J.H**. Writing – review & editing: **N.Z**.

## Conflicts of Interest

The authors declare no conflicts of interest.

## Supporting information




**Supporting File 1**: advs77352‐sup‐0001‐SuppMat.docx.

**Supporting File 2**: advs77352‐sup‐0002‐MovieS1.avi.

**Supporting File 3**: advs77352‐sup‐0003‐MovieS2.avi.

**Supporting File 4**: advs77352‐sup‐0004‐MovieS3.avi.

**Supporting File 5**: advs77352‐sup‐0005‐MovieS4.avi.

**Supporting File 6**: advs77352‐sup‐0006‐MovieS5.avi.

**Supporting File 7**: advs77352‐sup‐0007‐MovieS6.avi.

## Data Availability

All data needed to evaluate the conclusions in the paper are present in the paper and/or the Supplementary Materials

## References

[1] N. Miyamoto , L. D. Pham , J. H. Seo , K. W. Kim , E. H. Lo , and K. Arai , “Crosstalk between Cerebral Endothelium and Oligodendrocyte,” Cellular and Molecular Life Sciences 71 (2014): 1055–1066. 24132511 10.1007/s00018-013-1488-9 PMC3944198

[2] A. L. Cattin , J. J. Burden , L. Van Emmenis , et al., “Macrophage‐Induced Blood Vessels Guide Schwann Cell‐Mediated Regeneration of Peripheral Nerves,” Cell 162 (2015): 1127–1139. 26279190 10.1016/j.cell.2015.07.021 PMC4553238

[3] J. Huang , J. Li , S. Li , et al., “Netrin‐1–Engineered Endothelial Cell Exosomes Induce the Formation of Pre‐Regenerative Niche to Accelerate Peripheral Nerve Repair,” Science Advances 10 (2024): adm8454. 10.1126/sciadv.adm8454 PMC11212737 38941462

[4] J. M. Gomez‐Salinero and S. Rafii , “Endothelial Cell Adaptation in Regeneration,” Science 362 (2018): 1116–1117. 30523098 10.1126/science.aar4800

[5] N. Saiki , Y. Nio , Y. Yoneyama , et al., “Self‐Organization of Sinusoidal Vessels in Pluripotent Stem Cell‐Derived Human Liver Bud Organoids,” Nature biomedical engineering 9 (2025): 1869–1885. 10.1038/s41551-025-01416-6 PMC12486625 40562862

[6] H. G. Augustin and G. Y. Koh , “Organotypic Vasculature: From Descriptive Heterogeneity to Functional Pathophysiology,” Science 357 (2017): aal2379. 10.1126/science.aal2379 28775214

[7] G. Li , R. Craig‐Schapiro , D. Redmond , et al., “Vascularization of Human Islets by Adaptable Endothelium for Durable and Functional Subcutaneous Engraftment,” Science Advances 11 (2025): adq5302. 10.1126/sciadv.adq5302 PMC11777203 39879286

[8] R. Wasko , K. Bridges , R. Pannone , et al., “Langerhans Cells are Essential Components of the Angiogenic Niche during Murine Skin Repair,” Developmental Cell 57 (2022): 2699–2713. 36493773 10.1016/j.devcel.2022.11.012 PMC10848275

[9] G. P. Bhat , A. Maurizio , A. Motta , et al., “Structured Wound Angiogenesis Instructs Mesenchymal Barrier Compartments in the Regenerating Nerve,” Neuron 112 (2024): 209–229. 37972594 10.1016/j.neuron.2023.10.025

[10] C. Li , H. Zhao , Z. Liu , and C. McMahon , “Deer Antler—A Novel Model for Studying Organ Regeneration in Mammals,” The International Journal of Biochemistry & Cell Biology 56 (2014): 111–122. 25046387 10.1016/j.biocel.2014.07.007

[11] Q. Guo , G. Zhang , J. Ren , et al., “Systemic Factors Associated with Antler Growth Promote Complete Wound Healing,” NPJ Regenerative Medicine 10 (2025): 4. 39833274 10.1038/s41536-025-00391-5 PMC11756403

[12] T. Qin , G. Zhang , Y. Zheng , et al., “A Population of Stem Cells with Strong Regenerative Potential Discovered in Deer Antlers,” Science 379 (2023): 840–847. 36821675 10.1126/science.add0488

[13] Y. Hao , B. Yu , M. Qin , et al., “Extracellular Vesicles from Antler Blastema Progenitor Cells Reverse Bone Loss and Mitigate Aging‐Related Phenotypes in Mice and Macaques,” Nature Aging 5 (2025): 1790–1809. 40660003 10.1038/s43587-025-00918-x PMC12443635

[14] H. Li , Y. Liu , Y. Lin , et al., “Cardiac Repair using Regenerating Neonatal Heart Tissue‐Derived Extracellular Vesicles,” Nature Communications 16 (2025): 1292. 10.1038/s41467-025-56384-x PMC11790877 39900896

[15] C. de Boer and N. H. Davies , “Blood Derived Extracellular Vesicles as Regenerative Medicine Therapeutics,” Biochimie 196 (2022): 203–215. 34688790 10.1016/j.biochi.2021.10.009

[16] W. Wang , Q. Guo , and C. Li , “Local and Systemic Factors both Required for Full Renewal of Deer Antlers, and Systemic Factors only for Generic Cutaneous Regenerative Healing,” Cell Regeneration 14 (2025): 24. 40493325 10.1186/s13619-025-00233-1 PMC12151924

[17] R. Zuo , Q. Liao , Z. Ye , et al., “Antler Blood Enhances the Ability of Stem Cell‐Derived Exosomes to Promote Bone and Vascular Regeneration,” Regenerative therapy 26 (2024): 1168–1180. 39640919 10.1016/j.reth.2024.11.003 PMC11617708

[18] R. Craig‐Schapiro , G. Li , K. Chen , et al., “Single‐Cell Atlas of Human Pancreatic Islet and Acinar Endothelial Cells in Health and Diabetes,” Nature Communications 16 (2025): 1338. 10.1038/s41467-024-55415-3 PMC11802906 39915484

[19] K. Rohlenova , J. Goveia , M. García‐Caballero , et al., “Single‐Cell RNA Sequencing Maps Endothelial Metabolic Plasticity in Pathological Angiogenesis,” Cell Metabolism 31 (2020): 862–877. 32268117 10.1016/j.cmet.2020.03.009

[20] B. Liu , Y. Jin , J. Yang , et al., “Extracellular Vesicles from Lung Tissue Drive Bone Marrow Neutrophil Recruitment in Inflammation,” Journal of Extracellular Vesicles 11 (2022): 12223. 10.1002/jev2.12223 PMC9122834 35595717

[21] Y. Zhang and J. Han , “Differential Privacy Fuzzy C‐Means Clustering Algorithm based on Gaussian Kernel Function,” PLoS ONE 16 (2021): 0248737. 10.1371/journal.pone.0248737 PMC7987176 33755689

[22] P. Fichelson , C. Moch , K. Ivanovitch , et al., “Live‐Imaging of Single Stem Cells within Their Niche Reveals that a U3snoRNP Component Segregates Asymmetrically and is Required for Self‐Renewal in Drosophila,” Nature Cell Biology 11 (2009): 685–693. 19430468 10.1038/ncb1874

[23] R. Browaeys , W. Saelens , and Y. Saeys , “NicheNet: Modeling Intercellular Communication by Linking Ligands to Target Genes,” Nature Methods 17 (2020): 159–162. 31819264 10.1038/s41592-019-0667-5

[24] Q. Zeng , M. Mousa , A. S. Nadukkandy , et al., “Understanding Tumour Endothelial Cell Heterogeneity and Function from Single‐Cell Omics,” Nature Reviews Cancer 23 (2023): 544–564. 37349410 10.1038/s41568-023-00591-5

[25] M. Astone , R. E. Oberkersch , G. Tosi , A. Biscontin , and M. M. Santoro , “The Circadian Protein BMAL1 Supports Endothelial Cell Cycle during Angiogenesis,” Cardiovascular research 119 (2023): 1952–1968. 37052172 10.1093/cvr/cvad057

[26] J. Huan , X. Liu , N. Wang , Y. Mu , L. Li , and Y. Du , “The RRP9‐JUN Axis Promotes Breast Cancer Progression via the AKT Signalling Pathway,” Biology Direct 19 (2024): 131. 39702367 10.1186/s13062-024-00578-8 PMC11660783

[27] E. C. Lerner , K. I. Woroniecka , V. M. D'Anniballe , et al., “CD8^+^ T Cells Maintain Killing of MHC‐I‐Negative Tumor Cells Through the NKG2D–NKG2DL Axis,” Nature cancer 4 (2023): 1258–1272. 37537301 10.1038/s43018-023-00600-4 PMC10518253

[28] L. Ke , Q. He , J. Qu , et al., “Bone‐Protective Effects of Neutralizing Angiopoietin‐Like Protein 4 Monoclonal Antibody in Rheumatoid Arthritis,” Molecular Therapy 32 (2024): 4497–4513. 39367607 10.1016/j.ymthe.2024.09.031 PMC11638830

[29] A. S. Harney , G. S. Karagiannis , J. Pignatelli , et al., “The Selective Tie2 Inhibitor Rebastinib Blocks Recruitment and Function of Tie2^Hi^ Macrophages in Breast Cancer and Pancreatic Neuroendocrine Tumors,” Molecular Cancer Therapeutics 16 (2017): 2486–2501. 28838996 10.1158/1535-7163.MCT-17-0241 PMC5669998

[30] Y. C. Jang and H. Van Remmen , “Age‐Associated Alterations of the Neuromuscular Junction,” Experimental Gerontology 46 (2011): 193–198. 20854887 10.1016/j.exger.2010.08.029 PMC3026920

[31] J. Kalucka , L. de Rooij , J. Goveia , et al., “Single‐Cell Transcriptome Atlas of Murine Endothelial Cells,” Cell 180 (2020): 764–779. 32059779 10.1016/j.cell.2020.01.015

[32] A. I. McDonald , A. S. Shirali , R. Aragón , et al., “Endothelial Regeneration of Large Vessels Is a Biphasic Process Driven by Local Cells With Distinct Proliferative Capacities,” Cell Stem Cell 23 (2018): 210–225. 30075129 10.1016/j.stem.2018.07.011 PMC6178982

[33] I. Hwang , E. J. Lee , H. Park , et al., “Endothelin‐1 Enhances the Regenerative Capability of Human Bone Marrow‐Derived Mesenchymal Stem Cells in a Sciatic Nerve Injury Mouse Model,” Biomaterials 275 (2021): 120980. 34198163 10.1016/j.biomaterials.2021.120980

[34] L. E. Olson and P. Soriano , “Increased PDGFRα Activation Disrupts Connective Tissue Development and Drives Systemic Fibrosis,” Developmental Cell 16 (2009): 303–313. 19217431 10.1016/j.devcel.2008.12.003 PMC2664622

[35] J. J. Ohab , S. Fleming , A. Blesch , and S. T. Carmichael , “A Neurovascular Niche for Neurogenesis After Stroke,” The Journal of Neuroscience 26 (2006): 13007–13016. 17167090 10.1523/JNEUROSCI.4323-06.2006 PMC6674957

[36] C. Dray , G. Rougon , and F. Debarbieux , “Quantitative Analysis by In Vivo Imaging of the Dynamics of Vascular and Axonal Networks in Injured Mouse Spinal Cord,” Proceedings of the National Academy of Sciences 106 (2009): 9459–9464. 10.1073/pnas.0900222106 PMC2685250 19470644

[37] R. Shechter and M. Schwartz , “CNS Sterile Injury: Just Another Wound Healing?,” Trends in Molecular Medicine 19 (2013): 135–143. 23279948 10.1016/j.molmed.2012.11.007

[38] J. Goveia , K. Rohlenova , F. Taverna , et al., “An Integrated Gene Expression Landscape Profiling Approach to Identify Lung Tumor Endothelial Cell Heterogeneity and Angiogenic Candidates,” Cancer Cell 37 (2020): 21–36. 31935371 10.1016/j.ccell.2019.12.001

[39] C. Han , M. Barakat , and L. A. DiPietro , “Angiogenesis in Wound Repair: Too Much of a Good Thing?,” Cold Spring Harbor Perspectives in Biology 14 (2022): a041225. 35667793 10.1101/cshperspect.a041225 PMC9524283

[40] L. MacCarthy‐Morrogh and P. Martin , “The Hallmarks of Cancer are also the Hallmarks of Wound Healing,” Science Signaling 13 (2020): aay8690. 10.1126/scisignal.aay8690 32900881

[41] A. Subramanian , P. Zakeri , M. Mousa , et al., “Angiogenesis Goes Computational—The Future Way Forward to Discover New Angiogenic Targets?,” Computational and Structural Biotechnology Journal 20 (2022): 5235–5255. 36187917 10.1016/j.csbj.2022.09.019 PMC9508490

[42] J. Malchow , J. Eberlein , W. Li , B. M. Hogan , K. S. Okuda , and C. S. M. Helker , “Neural Progenitor–Derived Apelin Controls Tip Cell Behavior and Vascular Patterning,” Science Advances 10 (2024): adk1174. 10.1126/sciadv.adk1174 PMC11225789 38968355

[43] L. Han , F. Li , H. Wu , et al., “Targeting FABP4 to Inhibit AGEs ‐ RAGE / NF ‐ κB Signalling Effectively Ameliorates Nucleus Pulposus Dysfunction and Angiogenesis in Obesity‐Related Intervertebral Disc Degeneration,” Cell Proliferation 58 (2025): 70021. 10.1111/cpr.70021 PMC12414640 40090836

[44] M. F. Zhang , J. H. Wang , S. Sun , et al., “Catalpol Attenuates Ischemic Stroke by Promoting Neurogenesis and Angiogenesis via the SDF–1α/CXCR4 Pathway,” Phytomedicine 128 (2024): 155362. 38522312 10.1016/j.phymed.2024.155362

[45] R. Janssens , S. Struyf , and P. Proost , “The Unique Structural and Functional Features of CXCL12,” Cellular & Molecular Immunology 15 (2018): 299–311. 29082918 10.1038/cmi.2017.107 PMC6052832

[46] A. Caprioli , H. Zhu , and T. N. Sato , “CRBP‐III:LacZ Expression Pattern Reveals a Novel Heterogeneity of Vascular Endothelial Cells,” Genesis 40 (2000): 139–145. 10.1002/gene.20075 15493015

[47] J. Guo , X. Ma , D. Liu , et al., “A Distinct Subset of Urothelial Cells with Enhanced EMT Features Promotes Chemotherapy Resistance and Cancer Recurrence by Increasing COL4A1‐ITGB1 Mediated Angiogenesis,” Drug Resistance Updates 76 (2024): 101116. 38968684 10.1016/j.drup.2024.101116

[48] Q. Guo , D. Wang , Z. Liu , and C. Li , “Effects of p21 Gene Down‐Regulation Through RNAi on Antler Stem Cells In Vitro,” PLoS ONE 10 (2015): 0134268. 10.1371/journal.pone.0134268 PMC4550451 26308075

[49] D. Wang , D. Berg , H. Ba , H. Sun , Z. Wang , and C. Li , “Deer Antler Stem Cells are a Novel Type of Cells that Sustain Full Regeneration of a Mammalian Organ—Deer Antler,” Cell Death & Disease 10 (2019): 443. 31165741 10.1038/s41419-019-1686-y PMC6549167

[50] X. Rong , G. Zhang , Y. Yang , et al., “Transplanted Antler Stem Cells Stimulated Regenerative Healing of Radiation‐induced Cutaneous Wounds in Rats,” Cell Transplantation 29 (2020): 963689720951549. 32907381 10.1177/0963689720951549 PMC7784515

[51] J. Galipeau and L. Sensébé , “Mesenchymal Stromal Cells: Clinical Challenges and Therapeutic Opportunities,” Cell Stem Cell 22 (2018): 824–833. 29859173 10.1016/j.stem.2018.05.004 PMC6434696

[52] N. E. Sharpless and R. A. DePinho , “How Stem Cells Age and Why This Makes us Grow Old,” Nature Reviews Molecular Cell Biology 8 (2007): 703–713. 17717515 10.1038/nrm2241

[53] A. W. Seifert , S. G. Kiama , M. G. Seifert , J. R. Goheen , T. M. Palmer , and M. Maden , “Skin Shedding and Tissue Regeneration in African Spiny Mice (Acomys),” Nature 489 (2012): 561–565. 23018966 10.1038/nature11499 PMC3480082

[54] H. D. Zomer and A. G. Trentin , “Skin Wound Healing in Humans and Mice: Challenges in Translational Research,” Journal of Dermatological Science 90 (2018): 3–12. 29289417 10.1016/j.jdermsci.2017.12.009

[55] D. S. Foster , M. Januszyk , K. E. Yost , et al., “Integrated Spatial Multiomics Reveals Fibroblast Fate during Tissue Repair,” Proceedings of the National Academy of Sciences 118 (2021): 2110025118. 10.1073/pnas.2110025118 PMC8521719 34620713

[56] C. Li and J. M. Suttie , “Histological Studies of Pedicle Skin Formation and Its Transformation to Antler Velvet in Red Deer (Cervus Elaphus),” The Anatomical Record 260 (2000): 62–71. 10967537 10.1002/1097-0185(20000901)260:1<62::AID-AR70>3.0.CO;2-4

[57] X. Rong , W. Chu , H. Zhang , et al., “Antler Stem Cell‐Conditioned Medium Stimulates Regenerative Wound Healing in Rats,” Stem Cell Research & Therapy 10 (2019): 326. 31744537 10.1186/s13287-019-1457-9 PMC6862758

[58] G. Zhang , D. Wang , J. Ren , et al., “Antler Stem Cell‐Derived Exosomes Promote Regenerative Wound Healing via Fibroblast‐to‐Myofibroblast Transition Inhibition,” Journal of biological engineering 17 (2023): 67. 37940994 10.1186/s13036-023-00386-0 PMC10633995

[59] C. Li , C. G. Mackintosh , S. K. Martin , and D. E. Clark , “Identification of Key Tissue Type for Antler Regeneration Through Pedicle Periosteum Deletion,” Cell and Tissue Research 328 (2007): 65–75. 17120051 10.1007/s00441-006-0333-y

[60] T. Hongu , M. Pein , J. Insua‐Rodríguez , et al., “Perivascular Tenascin C Triggers Sequential Activation of Macrophages and Endothelial Cells to Generate a Pro‐metastatic Vascular Niche in the Lungs,” Nature Cancer 3 (2022): 486–504. 35469015 10.1038/s43018-022-00353-6 PMC9046090

[61] P. Umbarkar , S. Ejantkar , S. Y. Ruiz Ramirez , et al., “Cardiac Fibroblast GSK‐3α Aggravates Ischemic Cardiac Injury by Promoting Fibrosis, Inflammation, and Impairing Angiogenesis,” Basic Research in Cardiology 118 (2023): 35. 37656238 10.1007/s00395-023-01005-1 PMC11340261

[62] C. L. Hong , I. S. Yu , C. H. Pai , et al., “CD248 Regulates Wnt Signaling in Pericytes to Promote Angiogenesis and Tumor Growth in Lung Cancer,” Cancer Research 82 (2022): 3734–3750. 35950912 10.1158/0008-5472.CAN-22-1695

[63] H. Zhang , L. Wang , J. Cui , et al., “Maintaining Hypoxia Environment of Subchondral Bone Alleviates Osteoarthritis Progression,” Science Advances 9 (2023): abo7868. 10.1126/sciadv.abo7868 PMC10075992 37018403

[64] S. Li , Y. Yang , B. Yu , et al., “A Novel Deer Antler‐Inspired Bone Graft Triggers Rapid Bone Regeneration,” Advanced Materials 37 (2025): 2411571. 39707695 10.1002/adma.202411571 PMC11817900

[65] S. Ojha , S. Malla , and S. M. Lyons , “snoRNPs: Functions in Ribosome Biogenesis,” Biomolecules 10 (2020): 783. 32443616 10.3390/biom10050783 PMC7277114

[66] S. C. Chandrasekharappa , J. H. Smith , and G. L. Eliceiri , “Biosynthesis of Small Nuclear RNAs in Human Cells,” Journal of Cellular Physiology 117 (1983): 169–174. 6195166 10.1002/jcp.1041170206

[67] S. C. Tao , T. Yuan , B. Y. Rui , Z. Z. Zhu , S. C. Guo , and C. Q. Zhang , “Exosomes Derived from Human Platelet‐Rich Plasma Prevent Apoptosis Induced by Glucocorticoid‐Associated Endoplasmic Reticulum Stress in rat Osteonecrosis of the Femoral Head via the Akt/Bad/Bcl‐2 Signal Pathway,” Theranostics 7 (2017): 733–750. 28255363 10.7150/thno.17450 PMC5327646

[68] J. Wu , Y. Piao , Q. Liu , and X. Yang , “Platelet‐Rich Plasma‐Derived Extracellular Vesicles: A Superior Alternative in Regenerative Medicine?,” Cell proliferation 54 (2021): 13123. 10.1111/cpr.13123 PMC8666280 34609779

[69] S. Rui , L. Dai , X. Zhang , et al., “Exosomal miRNA‐26b‐5p From PRP Suppresses NETs by Targeting MMP‐8 to Promote Diabetic Wound Healing,” Journal of Controlled Release 372 (2024): 221–233. 38909697 10.1016/j.jconrel.2024.06.050

[70] Y. Xia , J. Zhang , G. Liu , and J. Wolfram , “Immunogenicity of Extracellular Vesicles,” Advanced Materials 36 (2024): 2403199. 10.1002/adma.202403199 38932653

[71] Y. Hao , B. Yu , M. Qin , et al., “Extracellular Vesicles from Antler Blastema Progenitor Cells Reverse Bone Loss and Mitigate Aging‐Related Phenotypes in Mice and Macaques,” Nature Aging 5 (2025): 1790–1809. 40660003 10.1038/s43587-025-00918-x PMC12443635

[72] A. K. Gupta , T. Wang , J. A. Rapaport , and M. Talukder , “Therapeutic Potential of Extracellular Vesicles (Exosomes) Derived From Platelet‐Rich Plasma: A Literature Review,” Journal of Cosmetic Dermatology 24 (2025): 16709. 10.1111/jocd.16709 PMC11845942 39618056

[73] X. Chen , Y. Luo , Q. Zhu , et al., “Small Extracellular Vesicles from Young Plasma Reverse Age‐Related Functional Declines by Improving Mitochondrial Energy metabolism,” Nature Aging 4 (2024): 814–838. 38627524 10.1038/s43587-024-00612-4 PMC11186790

[74] S. Jin , C. F. Guerrero‐Juarez , L. Zhang , et al., “Inference and Analysis of Cell‐Cell Communication using CellChat,” Nature Communications 12 (2021): 1088. 10.1038/s41467-021-21246-9 PMC7889871 33597522

[75] J. Zhang , X. Zhang , C. Wang , et al., “Conductive Composite Fiber with Optimized Alignment Guides Neural Regeneration Under Electrical Stimulation,” Advanced Healthcare Materials 10 (2021): 2000604. 10.1002/adhm.202000604 33300246

[76] M. Dong , B. Shi , D. Liu , et al., “Conductive Hydrogel for a Photothermal‐Responsive Stretchable Artificial Nerve and Coalescing with a Damaged Peripheral Nerve,” ACS Nano 14 (2020): 16565–16575. 33025785 10.1021/acsnano.0c05197

