# Supplementary material for: Pre‐Regenerative Endothelial Cells Empowered by Antler Blood Exosomes Orchestrate Peripheral Nerve Repair
Source: Adv Sci (Weinh). 2026 Aug 27:e22170. Online ahead of print. doi: 10.1002/advs.202522170 (PMC13521176; doi:10.1002/advs.202522170)
Supplement: Supplementary file 1 [file ADVS-9999-e22170-s007.docx]

Supplementary Information for

**Pre-regenerative endothelial cells empowered by antler blood exosomes orchestrate peripheral nerve repair**

Jinsheng Huang^1,2,#^, Qiang Chen^1,#^, Aihua Jin^3,#^, Xiaoqi Yang^1^, Hui Zhou^1^, Weiquan Gong^4^, Jin Cheng^5^, Jun Dai^6^, Minjin Wang^7^, Meihua Piao^3,*^, Yuanyi Wang^4,*^, Nan Zhou^1,*^

1. Department of Orthopedics, The First Affiliated Hospital of Zhengzhou University, Zhengzhou University, Zhengzhou 450052, China;
2. Department of Sports Medicine of the Second Affiliated Hospital, and Liangzhu Laboratory, Zhejiang University School of Medicine, Hangzhou 310000, China;
3. Department of Clinical Laboratory, Yanbian University Hospital, Yanji 133000, China;
4. Department of Spinal Surgery, The First Hospital of Jilin University, Orthopedics Center, Jilin University, Changchun 130021, China;
5. Department of Sports Medicine, Beijing Key Laboratory of Sports Injuries, Peking University Third Hospital, Beijing 100191, China;
6. Department of Orthopedic Surgery, The Second Affiliated Hospital of Soochow University, Suzhou 215000, China;
7. Department of Laboratory Medicine, West China Hospital of Sichuan University, Chengdu 610000, China;

\# These authors contributed equally: Jinsheng Huang, Qiang Chen, Aihua Jin

Corresponding authors:

Nan Zhou: [fcczhoun@zzu.edu.cn](mailto:fcczhoun@zzu.edu.cn)

Yuanyi Wang: [wangyuanyi@jlu.edu.cn](mailto:wangyuanyi@jlu.edu.cn)

Meihua Piao: [9000002542@ybu.edu.cn](mailto:9000002542@ybu.edu.cn)

**The file includes:**

Supplementary Materials and Methods

Figs. SI1 to SI24

Legends for movies S1 to S6

**Other Supplementary Material for this manuscript includes the following:**

Movies S1 to S6

**MATERIALS AND METHODS
Cell culture**

Human umbilical vein endothelial cells (HUVECs, ATCC) were maintained in endothelial cell medium (ScienCell) supplemented with 10 % exosome-depleted fetal bovine serum (SBI), 1 % endothelial cell growth supplement (ECGS), and 1 % penicillin–streptomycin at 37 °C in a 5 % CO₂ humidified incubator. All functional assays were performed on cells between passages 4 and 6.

PC12 cells (Sunncell, China) were maintained in high-glucose Dulbecco's modified Eagle medium (DMEM; Gibco) supplemented with 10% exosome-depleted fetal bovine serum (FBS; SBI) and 1% penicillin-streptomycin (Gibco), and used between passages 4–6.

RSC96 rat Schwann cells (IMMOCELL, China) were cultured in DMEM (Gibco) containing 10% exosome-depleted FBS (SBI) and 1% penicillin-streptomycin (Gibco), and used between passages 4–6.

Primary rat dorsal root ganglion (DRG) neurons were isolated from Sprague-Dawley rats (postnatal days 1–3). Briefly, DRG were dissected in Hank's balanced salt solution and enzymatically digested with 0.25% trypsin at 37°C for 30 min, followed by mechanical dissociation through a fire-polished Pasteur pipette (<10 passes). Trypsinization was terminated with DMEM containing 10% fetal calf serum, and cells were pelleted by centrifugation. Dissociated neurons were resuspended, counted, and maintained in Neurobasal medium (Gibco) supplemented with 2% B27 (Gibco), 0.5% penicillin-streptomycin, and 50 ng/mL NGF (MedChemExpress).

**Tube formation assay**

Endothelial tube formation was assessed with the BD Endothelial Cell Tube Formation Assay. ECs were serum-starved for 24 h in medium containing 2 % FBS. A 96-well plate was coated with 50 μL Matrigel per well and polymerized for 1 h at 37 °C. ECs were seeded onto the gel and incubated for 6 and 12 h at 37 °C. Tubular networks were imaged by confocal microscopy, and junction numbers were quantified across groups.

**Scratch wound migration assay**

Endothelial migration was assessed by an in vitro scratch-wound assay. ECs were grown to ~90 % confluence in 6-well plates in complete medium. Then a uniform cross-shaped wound was created (time 0). After three PBS washes to remove debris and detached cells, the monolayers were maintained in serum-free ECM until full wound closure.

**Cell migration assay**

Cell migration was evaluated in 24-well Transwell inserts (8 μm pore, Corning, USA). A total of 1 × 10⁴ cells in 100 µL serum-free medium were seeded into the upper chamber, while 650 µL complete medium served as the chemoattractant in the lower chamber. After 24 h, non-migratory cells on the upper surface were removed with a cotton swab. The filters were washed with PBS, fixed with 4 % paraformaldehyde for 30 min, stained with 1 % crystal violet (Solarbio, China) for 30 min, rinsed again, and air-dried. Migrated cells on the underside were imaged under a phase-contrast microscope.

**Colony‑forming assay**

For colony-formation assays, 1000 cells per well were seeded in 6-well plates and cultured for 10 days. Colonies were fixed and stained with 0.1 % Crystal Violet solution, counted manually, and imaged under an inverted microscope to assess morphology across treatment groups.

**EdU staining assay**

Cell proliferation was additionally quantified with the 5-ethynyl-2'-deoxyuridine (EdU) Imaging Kits (K1075, APExBio, Houston, USA). After EdU incorporation and fixation, nuclei were counterstained with DAPI (Servicebio, China). EdU-positive cells were visualized under a light microscope (Olympus, Japan) and counted in five randomly selected fields per well; the percentage of proliferating cells was calculated relative to the total DAPI-stained nuclei.

**Co-culture system**

To investigate EC-nerve cell crosstalk, a cell culture insert (NEST Biotechnology Co. Ltd., China) co-culture system was established. Nerve cells were seeded in the upper chamber, while AB-EXO-treated ECs were plated in the lower compartment; migrated cells were subsequently stained and analyzed. In parallel, nerve cells were treated with anti-ANGPTL4 antibody (MedChemExpress) or rebastinib (MedChemExpress) in the upper chamber, with endothelial cells in the lower compartment, followed by migration analysis. Additionally, DRG neurons were cultured with conditioned medium from differentially treated endothelial cells, and axonal length was quantified.

**Blood Routine Examination and Serum Biochemical Indicators in Rats**

Whole blood was analyzed using an XN-series automated hematology analyzer. Hepatic and renal function parameters were measured using commercial assay kits (Rayto, China) on a specialized chemistry analyzer. Serum concentrations of interleukin-4 (IL-4), interleukin-10 (IL-10), interleukin-1β (IL-1β), and tumor necrosis factor-α (TNF-α) were quantified by chemiluminescence immunoassay.

**Histological staining**

The organs were excised, fixed overnight in 4 % PFA, dehydrated through graded ethanol, and paraffin-embedded. Five-micron cross- and longitudinal sections were de-paraffinized, rehydrated, and stained with hematoxylin–eosin (H&E) and Masson’s trichrome per manufacturer protocols. Slides were mounted with neutral resin and imaged on a light microscope.

**EXO treatment in vivo**

After PNI model establishment, rats were randomized into four groups: sham, PNI, AS-EXO, and AP-EXO. Immediately following PNI induction, the PNI group received 10 µL PBS, while the AS-EXO and AP-EXO groups received 10 µL of 100 µg mL⁻¹ AS-EXO or AP-EXO, respectively, delivered beneath the sciatic epineurium with a Hamilton micro syringe. The sham group underwent identical exposure without nerve injury or injection.

**Staining of Neuromuscular Junctions**

Muscle sections (8 μm thick) were washed with PBS and stained for Acetyl cholinesterase (AChE) using copper ferrocyanide method. For the immunofluorescent staining of NMJs, muscle sections (30 μm thick) were incubated for 60 minutes in blocking buffer and then incubated in mouse anti-NF200 (1:400; CST, USA) overnight at 4℃. The next day, sections were washed in PBS and incubated with appropriate fluorescently labeled secondary antibodies (1:1000; Abcam) for 1 hour at room temperature. In addition, endplates were stained with α-Bungarotoxin (1:1000, Biotium), which bind specifically to acetylcholine receptors (AChRs) in the postsynaptic membrane, thereby marking synaptic sites.

**Walking track analysis**

Motor recovery was assessed 28 days post-operation by walking-track analysis. The plantar surfaces of both hind paws were coated with Eosin Y (Solarbio), and each rat was encouraged to traverse a narrow, white-paper-lined corridor toward a darkened goal box. From the resulting footprints we measured, for both the normal (N) and experimental (E) limbs: paw length (PL), distance from toe 1 to toe 5 (toe spread, TS), and distance from toe 2 to toe 4 (intermediate toe spread, IT). The Sciatic Functional Index (SFI) was then calculated as:

$$\text{SFI=}\frac{\text{-38.3}\text{×}\left( \text{EPL-NPL} \right)}{\text{NPL}}\text{+}\frac{\text{109.5}\text{×}\left( \text{ETS-NTS} \right)}{\text{NTS}}\text{+}\frac{\text{13.3}\text{×}\left( \text{EIT-NIT} \right)}{\text{NIT}}\text{-}\text{8.8}$$

Walking-track analysis converts hind-limb footprint metrics into an index of neuromuscular function: an SFI of 0 denotes normal innervation, whereas −100 signifies total motor loss.

**Fig.SI-1 Cellular and molecular landscape of ECs within sciatic nerves in sham, 3 dpc, and 7 dpc groups.** a) Dot plot displaying marker gene expression across major cell classes. b) Percentages of different cell types. c) UMAP plot showing the distribution of all EC clusters. d) Violin plots displaying marker genes in different EC clusters. e) Relative density and distribution of AECs, NIECs, BECs and LECs in different groups.

**Fig.SI-2 Enrichment analysis of each EC cluster.** a) Seurat clustering analysis of G2/M and S scores in different EC clusters. b, c) GO term and KEGG enrichment analyses of specifically expressed genes in AECs, NIECs, BECs, and LECs.

**Fig.SI-3 Molecular landscape and pathways of AECs across groups.** a) Heatmap showing DEGs among sham, 3 dpc, and 7 dpc groups. b) Violin plots showing distribution of angiogenesis, exosome, nerve repair, and axon regeneration markers in different groups. c, d) GO term and KEGG enrichment analysis between different groups.

**Fig.SI-4 CellChat analysis of AECs, NIECs, BECs and LECs in different groups.** a) The total ligand-receptor (L-R) pair analysis among different groups. b) Dot plots showing the incoming and outgoing communication patterns of target cells in different groups. c) Network and heatmap plots displaying the number of cell interactions and interaction weights in different groups. d) Heatmaps illustrating the contributions of each EC cluster to the ANGPT and ANGPTL signaling pathways across different groups. e) ANGPT/ANGPTL L–R contributions by group.

**Fig.SI-5.** The expressions of exosome-specifc markers, including positive markers (CD63, CD81, TSG101) and negative markers (Calnexin) were analyzed by western blot.

**Fig.SI-6 AB-EXO promoted angiogenesis in vitro.** a, b) Cell-cycle profiling by flow cytometry with statistical summary. c, d) Analysis of EC migration capacity using transwell assay. Scale bar, 50 μm. e, f) Representative images of colony formation with statistical analysis. Scale bar, 500 μm. ns = not significant, **P* < 0.05, ***P* < 0.01, ****P* < 0.001, *****P* < 0.0001.

**Fig.SI-7.** **Functional comparison between AB-EXO and bovine serum exosomes (BS-EXO).** a,b) Wound healing rate of ECs in different groups after 12 h and 24 h. Scale bar, 100 μm. c,d) Analysis of EC migration capacity using transwell assay. Scale bar, 50 μm. e,f) Tube formation ability of ECs with each treatment at 12 h. Scale bar, 100 μm. **P < 0.01, ***P < 0.001.

**Fig.SI-8 Enrichment analysis for different clusters.** a) Domain, GO term and KEGG enrichment analysis for different clusters. b) Protein–protein interaction (PPI) network of differentially expressed molecules; up-regulated proteins in red, down-regulated in green.

**Fig.SI-9 Molecular landscape and enrichment analysis between IMP3+AECs and IMP3-AECs.** a) Relative density of IMP3 in EC clusters. b) Heatmap showing DEGs between IMP3+AECs and IMP3-AECs. c,d) GO term and KEGG enrichment analysis between IMP3+AECs and IMP3-AECs.

**Fig.SI-10 Pseudo-time analysis for IMP3+AECs and IMP3-AECs.** a) CytoTRACE analysis for IMP3+AECs and IMP3-AECs. b) Pseudo-time kinetics of representative genes. c, d) Cell trajectories by lineage state and pseudo-time among AECs. e) Hierarchical-clustering heat map displaying branch-specific gene-expression trajectories, grouped into six dynamic patterns.

**Fig.SI-11 CellChat analysis of IMP3+AECs, IMP3-AECs, NIECs, BECs and LECs.** a) The total L-R pair analysis among different groups. b) Dot plots showing the incoming and outgoing communication patterns of IMP3+AECs, IMP3-AECs, NIECs, BECs and LECs. c) ANGPT/ANGPTL/APELIN L-R contributions by group.

**Fig.SI-12 NicheNet analysis of IMP3+AECs, IMP3-AECs, NIECs, BECs and LECs.** a) Dot plots illustrating the predicted ligand expression profiles specifically within the IMP3+AEC population. b) Heatmaps depicting the regulatory strength of ligands toward their predicted target genes. c) Heatmaps quantifying the prior interaction potential between NicheNet-predicted ligands and their cognate receptors; intensity reflects predicted binding strength.

**Fig.SI-13 Verification of IMP3 transfection efficiency across LV-NC (negative control), LV-IMP3 (overexpression) and LV-shIMP3 (knockdown) groups.** a) Quantitative PCR analysis of IMP3 expression levels in ECs. b) Western blot confirming differential IMP3 protein levels among the LV-NC, LV-IMP3 and LV-shIMP3 groups.

**Fig.SI-14.** IMP3 knockdown ablates the regenerative effects of AB-EXO. a,c) Representative EdU staining images in different groups with statistical analysis. Scale bar, 100 μm. b,d) Analysis of EC migration capacity using transwell assay. Scale bar, 50 μm. ns = not significant, **P < 0.01, ***P < 0.001, ****P < 0.0001.

**Fig.SI-15.** Loss of IMP3 abrogates AB-EXO-mediated pro-neurogenic effects in co-culture. a) Schematic of the coculture system. b,c) Analysis of PC12 cell migration capacity using cell culture insert in a co-culture system. Scale bar, 50 μm. d,e) Analysis of RSC96 cell migration capacity using cell culture insert assay in co-culture system. Scale bar, 50 μm. f,g) Representative staining images of DRG neurons treated with different conditioned medium from LV-NC ECs, AS-EXO-treated LV-NC ECs, AP-EXO-treated LV-NC ECs, LV-shIMP3 ECs, AS-EXO-treated LV-shIMP3 ECs and AP-EXO-treated LV-shIMP3 ECs. Scale bar, 50 μm. ns = not significant, **P < 0.01, ***P < 0.001, ****P < 0.0001.

**Fig.SI-16.** Blockade of ANGPTL4-TEK signaling eliminates endothelial cell-mediated benefits to nerve cells. a) Schematic of the coculture system. b,c) Analysis of PC12 cell migration capacity using cell culture insert assay in co-culture system. Scale bar, 50 μm. d,e) Analysis of RSC96 cell migration capacity using cell culture insert assay in co-culture system. Scale bar, 50 μm. f,g) Representative immunofluorescence images of DRG neurons treated with anti-ANGPTL4 antibody and TEK inhibitor, followed by co-culture with conditioned medium from LV-NC or LV-IMP3 ECs. Scale bar, 50 μm. ns = not significant, ****P < 0.0001.

**Fig.SI-17.** A pre-regenerative vascular niche (PVN) was visualized via 3D reconstruction of the injury site. a,b) Fluorescence-based 3D reconstruction reveals vascular and axonal regeneration at 1, 3, and 7 days post crush (dpc). Scale bar, 50 μm. c,d) Representative 3D immunofluorescence images of injured sciatic nerves at 7 days post-crush (dpc) from rats treated with AAV-NC, AAV-shIMP3, or AAV-IMP3. Scale bar, 50 μm.

**Fig.SI-18. IMP3 promoted nerve regeneration.** **a**) Percentages of EGFP⁺ cells quantified by flow cytometry at 7, 14 and 28 days after PNI. **b**) Representative TEM images of sciatic nerve in each group. Scale bar, 1 μm.

**Fig.SI-19.** AB-EXO promoted nerve repair more effectively than BS-EXO. a) Representative H&E-stained cross- and longitudinal sections of sciatic nerves at 28 days post-surgery. Scale bar, 20 μm. b) Representative Loyez-stained sections of sciatic nerve. Scale bar, 20 μm. c,d) Representative quadruple immunofluorescence images (CD31/TuJ1/MBP/DAPI) of sciatic nerve cross- and longitudinal sections. Scale bar, 50 μm.

**Fig.SI-20.** IMP3 knockdown reversed the pro-regenerative effects of AB-EXO on injured nerves. a) Representative H&E stained cross and longitudinal-sections of sciatic nerves at 28 days post-surgery. Scale bar, 20 μm. b) Representative Loyez–stained sections of sciatic nerve. Scale bar, 20 μm. c) Representative quadruple immunofluorescence images (CD31/TuJ1/MBP/DAPI) of sciatic-nerve cross-sections. Scale bar, 50 μm. d) Representative immunofluorescence images of longitudinal sciatic-nerve sections. Scale bar, 50 μm.

**Fig.SI-21.** Local immune response in the sciatic nerve and systemic safety of AB-EXO. a,b) Representative images of CD68/iNOS/ARG1/ multiple immunofluorescence staining of sciatic nerve in different groups. Scale bar, 50 μm. c,d) The statistical results of percentages of iNOS and ARG1 positive staining area in different groups (n=5). e) The counts of WBC, RBC, PLT and HGB in the blood (n=5). f) Serum levels of ALT, AST, BUN and CREA (n=5). g) Serum level of inflammatory factors including IL10, IL-4, IL-1β and TNF-α (n = 5). h,i) H&E and Masson staining of major organs. Scale bar, 200 μm. ns = not significant, **P < 0.01, ****P < 0.0001

**Fig.SI-22.** AB-EXO outperformed BS-EXO in restoring nerve conduction and muscle re-innervation. a) Representative H&E- and Masson-stained cross-sections of gastrocnemius muscle. Scale bar, 20 μm. b) Quantification of mean muscle fiber diameter (n=5). c) Immunofluorescence staining of gastrocnemius NMJs. Scale bar, 100 μm. d) Quantification of innervated NMJ ratio (n=5). e) Representative immunofluorescence images of fast- and slow-myosin in gastrocnemius muscle cross-sections. Scale bar, 50 μm. f,g) Quantification of fast- and slow-type fiber percentages (n=5). ns = not significant, **P* < 0.05, ***P* < 0.01, ****P* < 0.001, *****P* < 0.0001

**Fig.SI-23.** IMP3 knockdown reverses AB-EXO–mediated functional recovery after nerve injury. a) Representative H&E and Masson-stained cross-sections of gastrocnemius muscle from each group. Scale bar, 20 μm. b) Quantification of the mean fiber diameter (n=5). c) Representative immunofluorescence images of fast-myosin and slow-myosin in gastrocnemius-muscle cross-sections. Scale bar, 50 μm. d,e) Quantification of the fast- and slow-type fiber percentages in gastrocnemius muscle (n=5). ns = not significant, **P* < 0.05, ***P* < 0.01, ****P* < 0.001, *****P* < 0.0001

**Fig.SI-24. The scheme reveals that antler blood-derived exosomes link IMP3 to the atlas of strong regenerative endothelial cells within the peripheral nerve.**

**Movie S1** Dynamic demonstration of 3D fluorescence imaging of the sciatic nerve in 1 dpc group.

**Movie S2** Dynamic demonstration of 3D fluorescence imaging of the sciatic nerve in 3 dpc group.

**Movie S3** Dynamic demonstration of 3D fluorescence imaging of the sciatic nerve in 7 dpc group.

**Movie S4** Dynamic demonstration of 3D fluorescence imaging of the sciatic nerve in AAV-NC group.

**Movie S5** Dynamic demonstration of 3D fluorescence imaging of the sciatic nerve in AAV-shIMP3 group.

**Movie S6** Dynamic demonstration of 3D fluorescence imaging of the sciatic nerve in AAV-IMP3 group.
